# Three-Dimensional Monte Carlo Simulation of PHBV/TPU Blend Biodisintegration Under Composting Conditions: Role of TPU Network Percolation

**DOI:** 10.3390/polym18192440

**Published:** 2026-10-07

**Authors:** Idoia San Sebastian, Gaizka Erkizia, Eva Rodriguez Hortelano, Maider Iturrondobeitia

**Affiliations:** 1 Department of Graphic Design and Engineering Projects, Bilbao School of Engineering, University of the Basque Country (EHU), 48013 Bilbao, Spain; eva.rodri@ehu.eus (E.R.H.); maider.iturrondobeitia@ehu.eus (M.I.); 2 Department of Graphic Design and Engineering Projects, Faculty of Engineering Gipuzkoa, University of the Basque Country (EHU), 20018 Donostia-San Sebastián, Spain; gaizka.erkizia@ehu.eus

**Keywords:** Monte Carlo simulation, PHBV, biodisintegration, composting, percolation, parameter identifiability, RSA morphology, enzymatic co-degradation

## Abstract

Blending poly(3-hydroxybutyrate-co-3-hydroxyvalerate) (PHBV) with thermoplastic polyurethane (TPU) improves toughness but compromises compostability above a critical TPU content. To our knowledge, this is the first three-dimensional, voxel-based Monte Carlo (MC) framework for simulating the disintegration of PHBV/TPU blends under ISO 20200 composting conditions. It couples Monod-type microbial growth, a cardinal-temperature correction, biomass and moisture transport, and a blend morphology generated by random sequential adsorption (RSA) and calibrated against scanning electron microscopy (SEM) data. The transition between complete and incomplete disintegration is described by partitioning the TPU phase into reachable and trapped fractions derived from the connectivity of the generated TPU network. Calibrated against eight compositions (100/0 to 0/100; 58 °C; 41 days) with 22 adjusted parameters, the model yields a root-mean-square error (RMSE) of 3.97% over all 40 fitted points and reproduces complete disintegration up to 40 wt% TPU, the incomplete disintegration of the 50/50 blend and the minimal degradation of neat TPU. In a hold-out test, the compositions withheld from calibration (90/10 and 70/30) were predicted with an RMSE of 3.23%. Sensitivity and identifiability analyses reveal a composition-dependent parameter hierarchy, and virtual temperature experiments illustrate the thermal behaviour implied by the model assumptions.

## 1. Introduction

Global production of fossil-based plastics reached 460 million metric tonnes in 2019 and continues to rise [1]. An estimated 4.8–12.7 million tonnes of plastic waste entered the ocean in 2010 alone [2], and plastic pollution and climate change are linked through mutually reinforcing feedbacks [3]. Plastic pollution is now recognised as exacerbating the impacts on all planetary boundaries, affecting terrestrial, aquatic and marine ecosystems [4]. Even polymers designed to biodegrade persist for periods that depend strongly on material, habitat and climate zone [5]. These concerns have driven the search for biodegradable alternatives derived from renewable resources, including blends that combine processability with end-of-life degradability [6]. Polyhydroxyalkanoates (PHAs) are a family of biopolymers, synthesised by microbial fermentation, that biodegrade under diverse conditions, including industrial composting, soil and aquatic environments [7,8]. Poly(3-hydroxybutyrate-co-3-hydroxyvalerate) (PHBV), a copolymer incorporating 3-hydroxyvalerate units to reduce crystallinity and improve processability, is attracting increasing attention for use in food packaging owing to its competitive barrier properties and complete biodegradability under industrial composting conditions [8,9].

**Figure 1:**
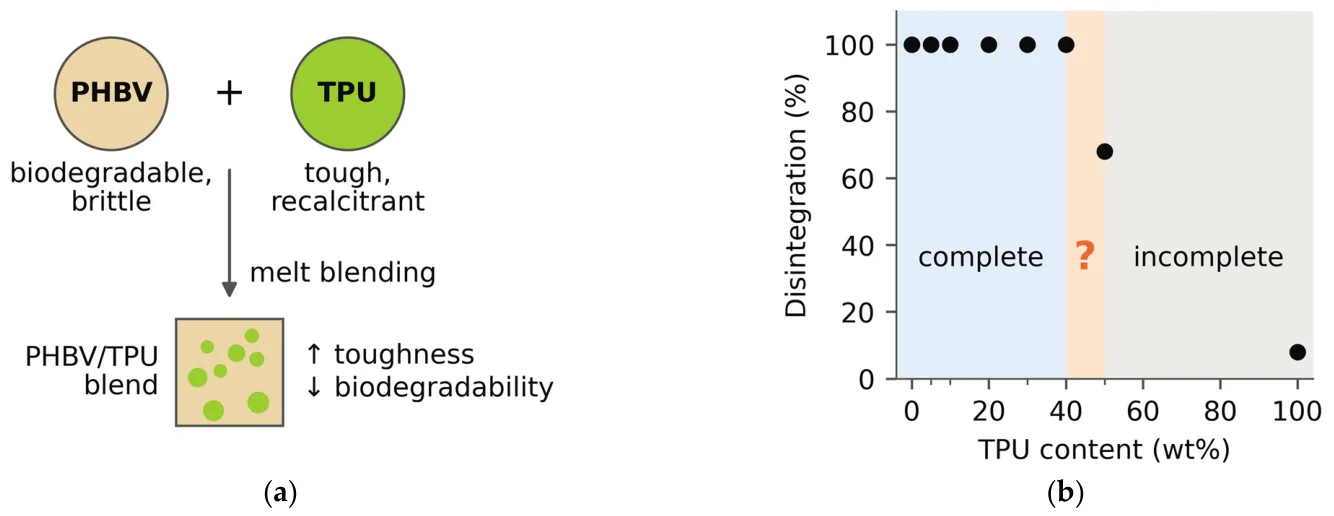
Motivation of the study. (**a**) Melt-blending poly(3-hydroxybutyrate-co-3-hydroxyvalerate) (PHBV) with thermoplastic polyurethane (TPU) improves toughness but reduces biodegradability. (**b**) Disintegration at day 41 under ISO 20200 as a function of TPU content, from Martínez-Abad et al. [14]; shaded areas: complete disintegration (blue), untested range (orange, marked "?") and incomplete disintegration (grey). Blends with ≤40 wt% TPU disintegrate completely, but the 50/50 blend does not, leaving the 40–50 wt% window unresolved.

However, the large-scale commercial adoption of PHBV is limited by its inherent brittleness, high crystallinity, narrow thermal processing window, and substantially higher production costs than conventional polymers [10,11]. These limitations have motivated melt blending with more flexible polymers [6]. Thermoplastic polyurethanes (TPUs) are segmented elastomers with a soft- and hard-block structure that gives them high elongation at break and resilience. They have been used as blending partners for PHBV [12,13]. Martínez-Abad et al. [14] reported that PHBV/TPU blends containing up to 30 wt% TPU exhibited improved barrier properties and substantially higher elongation at break. Subsequent studies have reported the effectiveness of this toughening strategy, including the use of reactive compatibilising agents [13,14,15]. In disintegration tests conducted in accordance with ISO 20200, all blends containing less than 50 wt% TPU (including the 60/40 blend) disintegrated completely by approximately day 36, following sigmoidal profiles. In contrast, the 50/50 blend reached only approximately 68% disintegration by day 41, with no tendency to plateau, and neat TPU remained at approximately 8%. The transition between complete and incomplete disintegration therefore lies at a TPU content between 40 and 50 wt% (Figure 1).

Understanding and predicting the biodegradation kinetics of these heterogeneous blends would support the rational design of formulations for the growing bioplastics market [16], reduce the dependence on costly and time-consuming composting experiments, whose outcomes vary considerably with incubation temperature, moisture and substrate composition [17,18], and help to elucidate spatiotemporal degradation mechanisms, such as enzymatic transport and the connectivity of the internal morphology, that are difficult to observe experimentally [19]. Bridging the gap between the controlled conditions of ISO 20200 at 58 °C and the degradation rates achieved in industrial facilities also remains a challenge [19,20], and mathematical modelling has been proposed as a means of addressing it [21].

Computational approaches to biodegradable and biobased polymers now span a range of scales, from quantum-level simulations to macroscale process modelling, and are increasingly used to complement, rather than replace, experimental characterisation [22]. The present work operates at the mesoscale (sub-micrometre voxels): no quantum-chemical, atomistic or molecular-dynamics calculations were performed, and molecular-level processes such as chain scission and enzyme–substrate binding enter the model only through effective event probabilities. Within this landscape, mathematical models of polymer biodegradation [21] have evolved considerably from early empirical kinetic and lattice-erosion approaches [23]. Göpferich developed continuum models that coupled reaction kinetics with diffusion [24], and Siepmann and Göpferich extended these models to incorporate chain scission, oligomer diffusion, and pH-dependent autocatalysis [25]. More recent frameworks have applied Monod kinetics to microbial growth in anaerobic digestion [26], applied first-order kinetics in series with a lag phase [27], formulated systems of nonlinear differential equations for poly(lactic acid) (PLA) degradation [28] and revealed substrate-repression effects in starch–polyolefin blends [29]. Other mechanistic approaches incorporate the polymer’s physicochemical structure. Gleadall et al. modelled random and terminal chain scission [30], Wang et al. proposed a phenomenological reaction–diffusion model [31], and Han and Pan extended this by coupling Avrami crystallisation theory with reaction–diffusion equations [32]. This coupling accounts for the preferential enzymatic attack on amorphous regions [33], which produces multi-stage profiles that simple kinetic functions do not capture.

Monte Carlo (MC) simulation methods have been applied to heterogeneous processes in which stochastic events govern material transformation [34], and they also allow uncertainty to be quantified [35]. At the molecular scale, they have been used to model chain scission dynamics [36,37], and multiscale frameworks have linked microscopic reactions to macroscopic diffusion [38]. Three-dimensional spatial resolution is required when the outcome is governed by transport limitations or the medium’s internal architecture, as in the autocatalytic acceleration produced by the accumulation of acidic degradation products inside an eroding matrix [30,31]. Lattice Monte Carlo schemes that resolve the enzymatic degradation of heterogeneous three-dimensional media have been developed in adjacent fields for this reason [39]. Mohammadi and Jabbari performed 3D MC simulations of poly(lactide) scaffold degradation [40], and Erkizia et al. implemented a Q-Potts MC algorithm with a resolution of 20 µm [41]. However, most existing MC models employ static transition probabilities, without coupling to composting microbial kinetics or to phase percolation [30,31,32,33,34,35,36,37,38,39,40,41,42]. To our knowledge, no framework combines three-dimensional spatial resolution with biologically grounded microbial growth kinetics, composition-dependent enzymatic mechanisms, and an explicit treatment of the percolation of the recalcitrant phase to simulate the biodisintegration of heterogeneous polymer blends under composting conditions.

This study addresses this gap by developing a three-dimensional, voxel-based Monte Carlo simulation framework. The framework couples Monod-type microbial growth kinetics with the cardinal temperature model with inflection (CTMI). It also incorporates coupled biomass and moisture transport through the lattice and a composition-dependent enzymatic co-degradation mechanism. In addition, it includes a percolation regime in which the trapped fraction of the TPU phase is derived from the connectivity of the generated morphology. The model was calibrated against experimental data from Martínez-Abad et al. [14], which cover all eight PHBV/TPU compositions (100/0, 95/5, 90/10, 80/20, 70/30, 60/40, 50/50 and 0/100) tested under ISO 20200 conditions at 58 °C for 41 days, and its predictive ability was tested on compositions withheld from calibration. A Morris elementary-effects sensitivity analysis was performed to establish the parameter hierarchy. Additionally, virtual temperature experiments were conducted to examine the thermal response implied by the CTMI correction.

## 2. Materials and Methods

The framework incorporates six elements within a single stochastic lattice model: material-specific Monod-type microbial growth kinetics; a temperature-dependent correction of the growth rate; heterogeneous PHBV/TPU morphology generated by random sequential adsorption (RSA); the explicit transport of biomass and moisture through the lattice; a set of concurrent degradation pathways for the TPU phase, including enzymatic co-degradation driven by PHBV matrix consumption; and a percolation regime, derived from the connectivity of the TPU phase, that determines which TPU voxels can be reached by co-degradation. All model symbols, together with their calibrated values and units, are listed in Table A1.

### 2.1. Spatial Discretisation and State Variables

**Figure 2:**
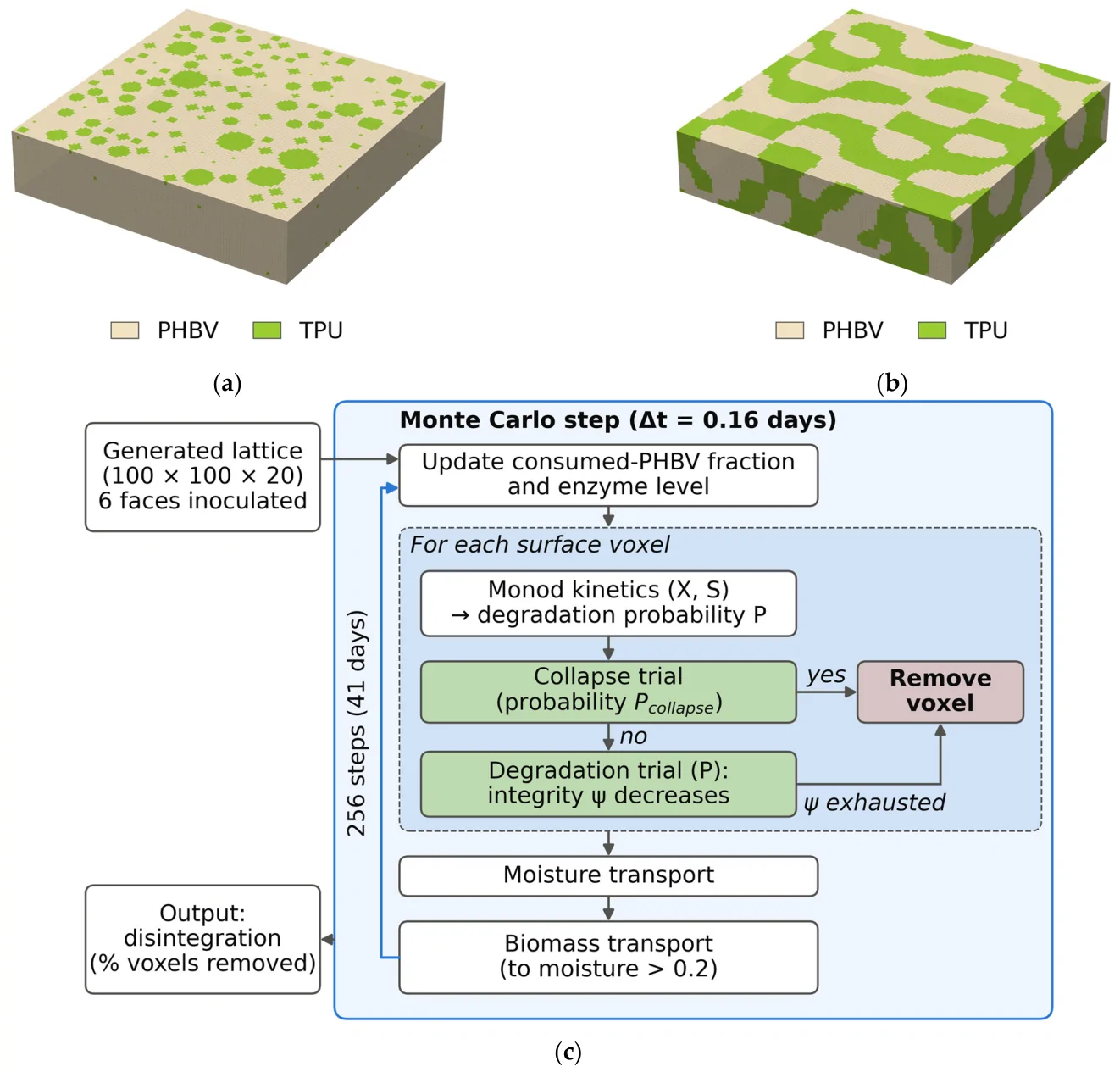
Model scheme. (**a**,**b**) Representative volume element (RVE) of 100 × 100 × 20 voxels at *t* = 0, as generated by the model (seed 1): (**a**) 80/20 blend, droplet-in-matrix morphology generated by random sequential adsorption (RSA); (**b**) 50/50 blend, co-continuous morphology. PHBV, cream; TPU, yellow green. (**c**) Sequence of operations in each Monte Carlo step (Δ*t* = 0.16 days), repeated 256 times to cover the 41-day test period.

The polymer specimen is represented as a three-dimensional, 100 × 100 × 20-voxel orthogonal lattice (200,000 voxels in total), which constitutes a representative volume element (RVE) in thin-film geometry with a 5:1 aspect ratio (Figure 2). Its occupancy is read at run time from an external geometry file with one row per voxel in (x, y, z, value) format; the lattice dimensions are inferred from the maximum indices, and a voxel is classified as polymer when its value exceeds 0.5. In the present work, the file describes a fully occupied rectangular block (200,000 polymer voxels); material identity (PHBV or TPU) is not read from the file but is assigned by the morphology generators. All neighbourhood operations (surface classification, local accessibility, transport and connectivity analysis) use the six face neighbours (von Neumann neighbourhood). The boundaries are non-periodic: the six external faces are in contact with the compost and act as inoculation surfaces. With a voxel edge of 0.2–0.4 µm, the simulated volume corresponds to 20–40 µm laterally and 4–8 µm in thickness.

Each voxel (i, j, k) carries six state variables: the occupancy state *φ* ∈ {0, 1}, which becomes 0 once the voxel is removed; the material identity *M* ∈ {PHBV, TPU}; the structural integrity ψ, initialised at 255 on an 8-bit scale and decremented in material-specific steps; the local biomass concentration *X* ∈ [0, K]; the local substrate fraction *S* ∈ [0, 1], initialised at 1.0 in every polymer voxel; and the local moisture *h* ∈ [0, 1], which gates the penetration of biomass into the interior of the film.

Structural integrity is lost progressively, in several steps. Each accepted degradation event decreases the integrity variable *ψ* by 10 units for PHBV and by 128 units for TPU. When the remaining integrity no longer exceeds the corresponding decrement, the voxel is removed (*φ* → 0). Therefore, a PHBV voxel requires up to 26 accepted events to be removed, whereas a TPU voxel requires only two. The integrity variable therefore resolves the gradual erosion of the matrix while treating the TPU phase as a two-state material. Collapse events, which apply to PHBV voxels only, bypass the integrity ladder and remove the voxel in a single step.

The voxel edge is set by matching the mean RSA domain diameter to the mean TPU domain diameter, as measured by scanning electron microscopy (SEM). This yields a voxel edge of 0.20 µm for the 90/10 blend and 0.40 µm for the 70/30 blend; depending on the composition, the voxel edge is approximately 0.2–0.4 µm. At this scale, the mean TPU domain spans four to ten voxels in diameter across the droplet-in-matrix compositions. The smallest domains permitted by the log-normal distribution span only two voxels.

The domain dimensions represent a compromise between morphological resolution and computational feasibility. Simulating the full experimental film thickness (200 µm) at the required resolution to visualise the TPU domains would require 500–1000 voxels in the *z* direction (5–10 million voxels in total), making the calibration, robustness and sensitivity campaigns impractical. However, the 100 × 100 × 20 lattice preserves the phase volume fractions, the SEM-calibrated domain size distribution, and the phase connectivity. These quantities control the degradation kinetics in the model, and the lattice resolves erosion fronts, biomass transport, and multi-step degradation. Three approximations follow from this choice and are assumed throughout. Firstly, the nominal weight fractions of the experimental blends are imposed on the lattice as volume fractions. Since the densities of PHBV and TPU differ by less than 15%, the resulting error in phase volume is also less than 15%. Secondly, the simulated film is thinner than the experimental one. Therefore, the model resolves the erosion front and its coupling to morphology rather than the absolute time required to traverse 200 µm of material. This difference is accounted for in the calibration of the kinetic parameters. Thirdly, the RVE is inoculated on all six of its external faces, so its lateral faces are more exposed than those of a macroscopic film specimen.

### 2.2. Generation of the Blend Morphology

Two morphology generators are used, depending on the composition. For PHBV fractions above 0.52, the blend is generated as a droplet-in-matrix (sea-island) morphology using the RSA algorithm. For PHBV fractions of 0.52 or below—that is, for TPU contents of 48 wt% or more—a co-continuous morphology is generated instead. Neat PHBV and neat TPU are assigned uniformly.

In the RSA generator, the lattice is initially set entirely to PHBV, and the required number of TPU voxels is calculated from the nominal TPU volume fraction. Spherical domains are then placed sequentially. The radius of each candidate domain is drawn from a log-normal distribution with a mean of $\bar{r}$ and a logarithmic dispersion of *σ*_ln_. This distribution is parameterised so that its arithmetic mean equals $\bar{r}$ and is truncated to a composition-specific interval [*r*_min_, *r*_max_] (Table 1). The domain centre is selected uniformly within the lattice, with the constraint that the sphere be fully contained in the *x* and *y* directions. In the *z* direction, however, the constraint is relaxed, requiring only that the centre lie inside the lattice. This allows domains to be truncated by the film surfaces, as observed experimentally. A candidate is rejected if its centre lies closer than *r_i_* + *r_j_* + 1 voxel to the centre of any previously placed domain, enforcing a minimum separation of one voxel between domain surfaces. Accepted domains convert every polymer voxel whose centre falls within the sphere to TPU. Placement continues until the target volume fraction is reached, with a maximum of 2 × 10^6^ attempts and 5000 domains.

Two provisions ensure that the target composition is met exactly. If the volume of a candidate sphere exceeds twice the remaining TPU volume, its radius is reduced so that the sphere’s volume is approximately three-quarters of the remaining volume. If 1000 consecutive placements are rejected because of overlap, the mean of the log-normal distribution is reduced by 5%. This enables the algorithm to continue filling a lattice that is becoming increasingly saturated. If the target is still not reached, the remaining TPU voxels are assigned individually at random to PHBV voxels that have no TPU face neighbour, so that the residual filling does not bridge neighbouring domains. This occurs for the 60/40 blend, where RSA placement saturates at about 32.5 vol%. The parameters in Table 1 are based on morphological evidence from three independent studies of PHBV/TPU blends [13,14,15].

The co-continuous generator superposes eight three-dimensional sinusoidal waves of the form sin(*fx*·*i* + *px*)·sin(*fy*·*j* + *py*)·sin(*fz*·*k* + *pz*), where the frequencies are uniformly distributed within the range [0.05, 0.35] rad·voxel^−1^, the phases are uniformly distributed within the range [0, 2π], and the amplitudes decay as 1/(1 + *w*) for the *w*th wave. The resulting scalar field is thresholded at a level determined by 50 bisection iterations so that the volume below this level is exactly equal to the target TPU fraction; voxels below this level are assigned to TPU, while the remainder are assigned to PHBV. This produces two interpenetrating phases without discrete domains, consistent with the reported morphology of PHBV/TPU at and above 50 wt% TPU.

After generation, the connectivity of the PHBV phase is audited by a breadth-first search over 6-connected PHBV voxels, seeded on the *z* = 0 face. This search determines whether the PHBV phase percolates through the film thickness and the proportion of PHBV voxels that belong to the spanning cluster. This audit characterises the generated morphology and does not enter the degradation kinetics.

### 2.3. Microbial Growth Kinetics and Thermal Correction

Microbial activity is resolved locally: each occupied voxel carries its own biomass and substrate, and the specific growth rate is evaluated for each voxel using Monod kinetics [43]:

(1) $$\mu =\mu_{max}\cdot \frac{S}{K_{S}+S}\cdot f\left(T\right)$$

where $\mu_{max}$x is the maximum specific growth rate, *K_S_* the half-saturation constant and *f*(*T*) the thermal factor. The three kinetic constants $\mu_{max}$, *K_S_* and the yield *Y* are material-specific, so that the TPU phase supports a slower and far less efficient microbial population than the PHBV phase.

The thermal factor uses the CTMI [44] to reproduce the asymmetric, non-Arrhenius response of microbial growth between minimum, optimum and maximum temperatures:

(2) $$f\left(T\right)=\frac{\left(T-T_{max}\right){\left(T-T_{min}\right)}^{2}}{\left(T_{opt}-T_{min}\right)\left[\left(T_{opt}-T_{min}\right)\left(T-T_{opt}\right)-\left(T_{opt}-T_{max}\right)\left(T_{opt}+T_{min}-2T\right)\right]}$$

with *T_min_* = 5 °C, *T_opt_* = 58 °C and *T_max_* = 70 °C, *f*(*T*) set to zero outside the open interval (*T_min_*, *T_max_*) and clipped to [0, 1]. At the ISO 20200 test temperature, *T* = *T_opt_* = 58 °C, and Equation (2) yields *f*(*T*) = 1, meaning that the thermal correction is inactive during calibration and only becomes active in virtual temperature experiments.

Growth is limited by a sigmoidal carrying-capacity factor that saturates the local biomass:

(3) $$f_{c}=\frac{1}{1+\exp\left[\kappa \left(\frac{X}{K}-\theta \right)\right]}$$

with *κ* = 4.0, *θ* = 0.85 and *K* = 1.0, clipped to [0, 1]. The biomass and substrate are then advanced by explicit Euler integration over one time step, Δ*t*:

(4) $$X\leftarrow X+\left(\mu -k_{d}\right)\cdot X\cdot f_{c}\cdot \Delta t$$

(5) $$\Delta S=\min\left[\frac{\left(\mu X\right)}{Y}\Delta t,S\right],S\leftarrow S-\Delta S$$

where $k_{d}$ = 0.0003 d^−1^ is the basal microbial death rate. Biomass is clipped to [0, *K*] and the substrate is floored at zero so that neither variable exceeds its physical range under the explicit scheme. A PHBV voxel whose substrate falls to 10^−4^ or below is treated as exhausted: Equations (1)–(5) are no longer updated for it, and its degradation probability is set to zero. This freezes both its biomass and residual substrate.

Because the inoculum is nine orders of magnitude below the carrying capacity, the growth stage itself sets the timescale of the lag phase. Integrating Equations (1)–(5) for a single surface voxel at 58 °C with the calibrated constants yields an enzymatic degradation probability of PHBV that becomes appreciable on day 15.2 and approaches saturation on day 17.6. The substrate falls below the collapse threshold on day 18.7. For a TPU voxel, however, the same integration does not reach the collapse threshold within 60 days: because of the lower $\mu_{max}$ and higher *K_S_* of TPU, the biomass would take more than 80 days to reach the threshold value. This asymmetry, a consequence of the material-specific Monod constants, means that neat TPU degrades by hydrolysis alone.

### 2.4. Degradation of the PHBV Phase

PHBV degradation is modelled as a biologically controlled erosion process. The probability that a PHBV voxel degrades in a Monte Carlo step is the sum of three contributions: an enzymatic term proportional to the actual substrate consumed in that step; a basal term representing sustained slow attack by an established population; and a substrate-depletion term with the same functional form as the collapse mechanism. The relevant equation is defined below:

(6) $$P_{deg,PHBV}=\Delta S\cdot \alpha_{MC}\cdot f_{c}+P_{basal}+P_{dep}$$

The first term converts the consumed substrate Δ*S* of Equation (5) into a removal probability through the Monte Carlo amplification factor $\alpha_{MC}$ = 20.0, which bridges the continuous kinetics and the discrete lattice event, and is attenuated by the same carrying-capacity factor that limits growth.

The basal term is gated by both biomass and time:

(7) $$P_{basal}=\left(b_{0}+b_{1}\cdot f_{b}\right)\cdot f_{t}\cdot f_{bu}\cdot g_{S}$$

with *b*_0_ = 0.001 and *b*_1_ = 0.004 per Monte Carlo step. The normalised biomass above the detection threshold *X_ε_* = 10^−6^ and the logistic activation switch are defined as follows:

(8) $$fb=\frac{X-X_{\varepsilon }}{K-X_{\varepsilon }},\;f_{bu}=\frac{1}{1+\exp\left[-100\left(X-X_{\varepsilon }\right)\right]}$$

with *fb* clipped to [0, 1]. With the values used here, the logistic factor $f_{bu}$ varies only between 0.5 (*X* = 0) and 1 (*X* = *K*), because the steepness of 100 is small relative to the unit scale of *X*; the condition $f_{bu}$ > 0.01, which the code requires before evaluating $P_{basal}$, is therefore always met, and $f_{bu}$ acts as a weak weighting factor rather than as a switch. The substrate gate is $g_{S}$ = 3.0 when the local substrate falls below 0.25 and $g_{S}$ = 1.0 otherwise, which accelerates the removal of nearly exhausted voxels. Finally, the factor

(9) $$f_{t}=\frac{1}{1+exp\left[-\kappa_{lag}\left(t-t_{lag}\right)\right]}$$

with $\kappa_{lag}$ = 0.01 d^−1^ and *t_lag_* = 15 days, varies only from 0.46 at day 0 to 0.56 at day 41 and is therefore nearly constant over the test. Consequently, $P_{basal}$ is of the order of 10^−4^–10^−3^ per Monte Carlo step on every surface PHBV voxel from the start of the test; because a PHBV voxel requires up to 26 accepted events to be removed, this contribution alone removes a negligible amount of material during the lag phase. The duration of the lag phase is set by the growth of the inoculum (Equations (1)–(5)) and by the moisture-gated penetration of biomass, not by ft or $f_{bu}$. Both factors are retained as implemented to keep the published code and the reported results consistent.

The third term, $P_{dep}$, is active when the local substrate falls below the collapse threshold $S_{c}$ and active biomass is present. It takes the following form:

(10) $$P_{dep}=r\cdot {\left(\frac{S_{c}-S}{S_{c}}\right)}^{2}\cdot \frac{X}{K}$$

with *r* = *r*_collapse,PHBV_. A term of the same form is also evaluated as an independent removal event in the main loop. The two are deliberately decoupled and may have different rate constants. The version entering Equation (6) decreases structural integrity, while the version evaluated in the main loop removes the voxel outright.

### 2.5. Degradation of the TPU Phase

**Figure 3:**
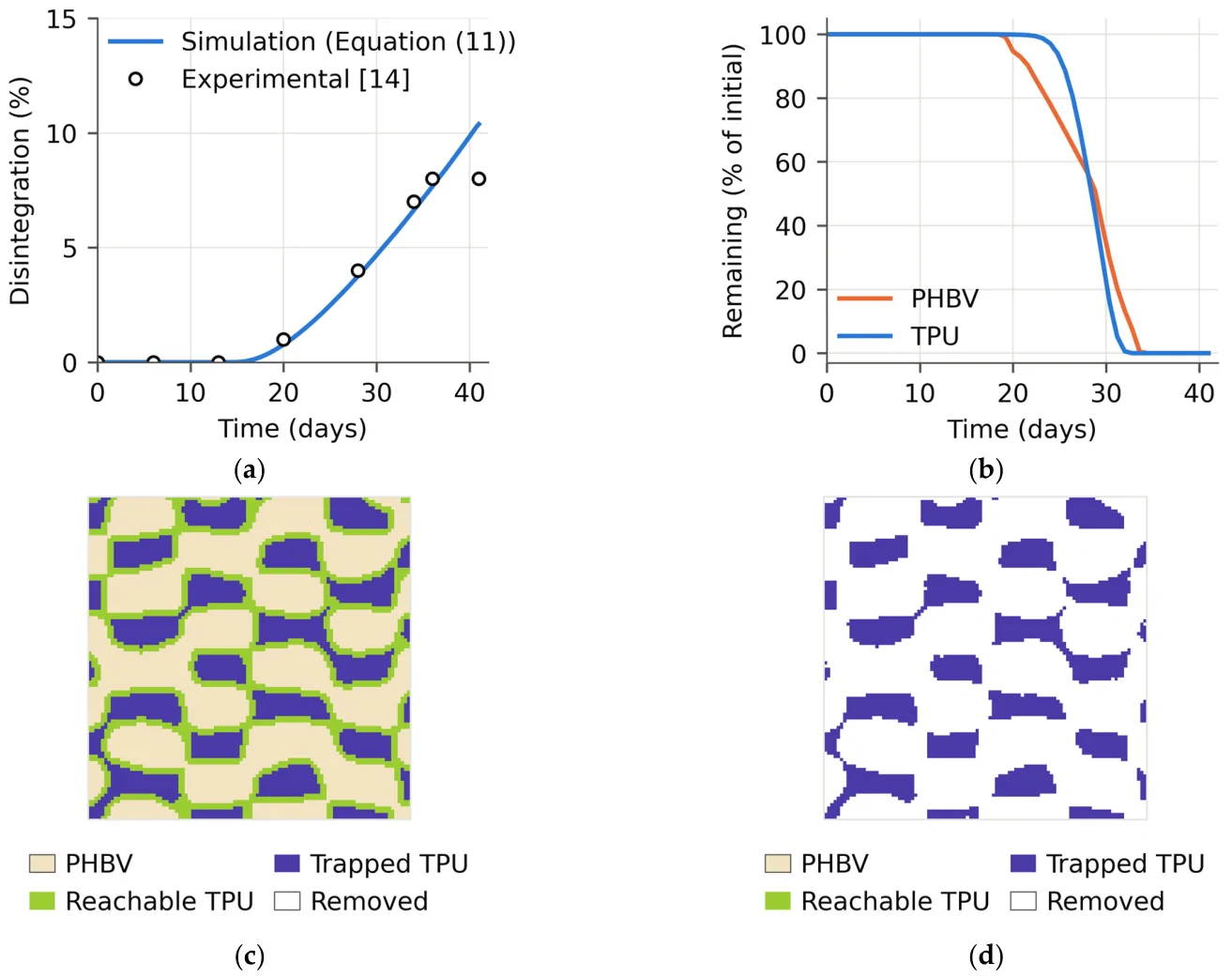
TPU degradation pathways and percolation regime in the model. (**a**) Neat TPU degrades only by abiotic hydrolysis (Equation (11)): simulation (mean and range of 20 replicates) and experimental data [14]. (**b**) In the 80/20 blend, TPU is removed by enzymatic co-degradation as the PHBV matrix is consumed (remaining fraction of each phase; mean of 20 replicates). (**c**) Mid-plane section of a generated 50/50 morphology at day 0: the TPU of the spanning network that lies more than δ = 2 voxels from the PHBV phase is trapped (violet), and the remaining TPU is reachable (green). (**d**) The same plane at day 41: PHBV and reachable TPU have been removed, whereas the trapped TPU persists, subject only to hydrolysis.

The probability of a TPU voxel degrading is built from three terms, the second of which is multiplicative and the others additive (Figure 3a,b).

(i) Abiotic hydrolysis. The base probability reflects the ester soft segment’s intrinsic susceptibility to hydrolytic scission [12,45] and is time-gated by its own logistic ramp:

(11) $$P_{hyd}\left(t\right)=\frac{p_{hyd}}{1+exp\left[-\kappa_{lag,TPU}\left(t-t_{lag,TPU}\right)\right]}$$

with $\kappa_{lag,TPU}$= 2.0 d^−1^ and $t_{lag,TPU}$= 15 days. Because this ramp is much steeper than that of Equation (9), hydrolysis starts within a few days around day 15 and then proceeds at a steady rate, which produces the quasi-linear disintegration profile of the 0/100 specimen. This pathway does not depend on *f*(*T*); this defines the biotic/abiotic separation examined in the virtual temperature experiments.

(ii) Local morphological accessibility. In blends, the accumulated probability is scaled by the local exposure of the voxel to the eroding matrix:

(12) $$P\leftarrow P\cdot {\left(\frac{n_{acc}}{6}\right)}^{m},\;m=1.2$$

where $n_{acc}$ is the number of the six face neighbours that have already been removed, are PHBV or lie outside the lattice, with a floor of 10^−5^ applied to the result. Therefore, a TPU voxel buried inside a large domain degrades far more slowly than one at the domain surface, introducing the surface-to-volume ratio of the domain size distribution directly into the kinetics. The co-degradation term (Equation (14)) is added after this scaling and is not attenuated by it.

The consumed PHBV fraction, pcf, tracks the cumulative degradation of the PHBV phase over the whole lattice:

(13) $$pcf=1-\frac{N_{PHBV}\left(t-\Delta t\right)}{N_{PHBV}\left(0\right)}$$

where *N_PHBV_* counts all PHBV voxels that have not been removed, whether they lie at the surface or in the interior.

(iii) Global enzymatic co-degradation. In the model, TPU removal in PHBV-rich blends is driven by the enzyme pool generated during PHBV consumption. The global enzyme level *E* is set to pcf (Equation (13)), and *p* = 2 is a front-advance exponent that delays the contribution of this pathway until an appreciable part of the matrix has been consumed:

(14) $$P\;\leftarrow \;P\;+\;r_{codeg}\;\cdot \;E^{p}\;\cdot \;f_{accessibility}$$

With

(15) $$f_{accessibility}=min\left(\sqrt{\frac{w_{TPU}}{0.35}},1\right)$$

where $w_{TPU}$ is the nominal TPU fraction. Once the remaining PHBV falls below 1% of its initial count, E is no longer refreshed and decays geometrically as *E* ← *λ*_E_·*E*, with *λ*_E_ = 0.95 per Monte Carlo step, representing the loss of extracellular enzyme activity when its inducing substrate is exhausted. The exponent *p* and the factor *λ*_E_ are fixed a priori.

Term (iii) acts only on the TPU voxels labelled as reachable by the percolation regime (Equation (18)), whereas hydrolysis (term *i*) acts on every TPU voxel. Unlike PHBV voxels, TPU voxels are not subject to substrate-depletion collapse.

### 2.6. Substrate-Depletion Collapse and Acceleration

A PHBV voxel whose local substrate has been depleted while active biomass is present may collapse and be removed in a single step, independently of the integrity-decrementing event. Collapse is evaluated when S < $S_{c}$ and X > X_min_, with $S_{c}$ = 0.50 and X_min_ = 0.01, and its probability is

(16) $$P_{collapse}=r\cdot {\left(\frac{S_{c}-S}{S_{c}}\right)}^{2}\cdot \frac{X}{K}$$

The quadratic dependence on the substrate deficit makes collapse negligible at the onset of depletion and dominant once the voxel is almost depleted, whereas the linear dependence on normalised biomass links the event to local microbial activity. The base rate is *r* = *r*_collapse,PHBV_.

The rate is modulated by an acceleration term, introduced to represent the sigmoidal shape of the experimental profiles. In a pure surface-erosion model, the number of active surface voxels remains approximately constant as the film erodes layer by layer. Therefore, the removal rate is quasi-linear. In contrast, the experimental profiles start slowly and accelerate towards completion. This is consistent with the progressive fragmentation of the film into ever smaller pieces. Visible fractures were reported from day 20 onwards, with the film breaking up into smaller pieces by day 28. The fragments were later smaller than 2 mm, and no visible fragments remained by day 36 [14]. Fragmentation multiplies the exposed surface area. The acceleration is written as follows:

(17) $$r\leftarrow r\cdot \left[1+A\cdot {\left(\frac{F_{deg}-a_{thr}}{1-a_{thr}}\right)}^{q}\right]$$

The acceleration is active only when the global degraded fraction of polymer, $F_{deg}$, exceeds the activation threshold $a_{thr}$ = 0.45; its intensity is A = 128 and the exponent is *q* = 2.0. Because the removal probability is bounded by one, large values of A make depleted surface voxels collapse almost immediately once $F_{deg}$ exceeds $a_{thr}$. For neat PHBV, *r* and A are scaled by two factors.

The collapse is evaluated as an independent Bernoulli trial before the integrity-decrementing events in Equations (6) and (11)–(14). This ensures that a voxel that collapses in a given step is not subjected to a second trial.

### 2.7. Percolation Regime and Neat-PHBV Kinetics

These two elements control, respectively, the transition between complete and incomplete disintegration and the kinetics of the matrix without a dispersed phase.

**Figure 4:**
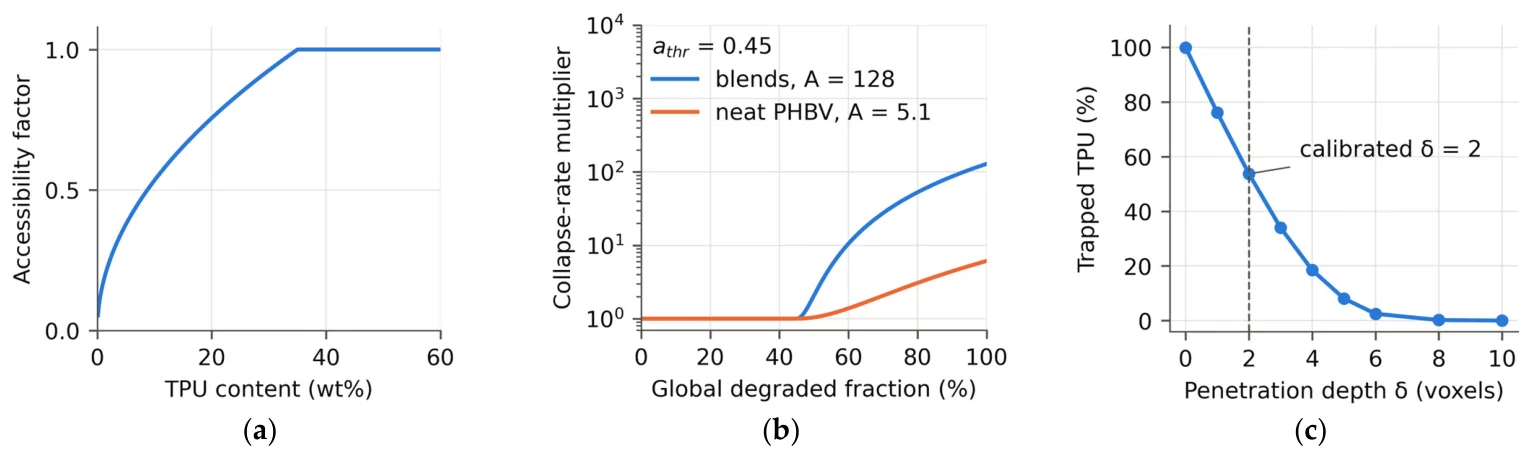
Composition-dependent functions of the model. (**a**) Enzymatic accessibility of TPU (Equation (15)), saturating at *w*_TPU_ = 35 wt%. (**b**) Multiplier of the collapse rate as a function of the global degraded fraction (Equation (17); $a_{thr}$ = 0.45), for the blends (A = 128) and neat PHBV (effective A = 5.1). (**c**) Fraction of the TPU trapped in the 50/50 blend as a function of the penetration depth δ (Equation (18); mean of five generated morphologies); blends with ≤ 40 wt% TPU contain no spanning TPU network and therefore no trapped TPU.

Experimentally, all blends with a TPU content below 50 wt% disintegrate completely, whereas the 50/50 blend only reaches 65% by day 36 and 68% by day 41 without completing the process [14]. In the model, the TPU voxels that the co-degradation pathway can reach are identified directly from the topology of the morphology generated for each realisation (Figure 3c). After generation, TPU clusters are labelled by a breadth-first search over 6-connected TPU voxels, and a cluster is classified as spanning when it connects opposite lateral faces of the lattice (the film thickness, 20 voxels, is comparable to the diameter of the largest droplets and is not used for this purpose). The distance of every TPU voxel to the nearest PHBV voxel, *d*_PHBV_, is computed by a multi-source breadth-first search through the TPU phase. A TPU voxel is labelled reachable if it belongs to a non-spanning cluster, since such domains are released as the surrounding matrix erodes, or if it lies within a penetration depth *δ* of the PHBV phase:

(18) $$reachable\;\Leftrightarrow \;\left(cluster\;not\;spanning\right)\;\vee \;\left(d_{PHBV}\;\leq \;\delta \right)$$

and trapped otherwise. The penetration depth, *δ* = 2 voxels, was calibrated against the 50/50 blend and is the only parameter of the percolation regime. Because the RSA generator enforces a minimum separation of one voxel between domains and the residual filling avoids bridging, TPU never forms a spanning network in the droplet-in-matrix compositions, and all of their TPU is reachable. In the co-continuous 50/50 morphology, 99.9% of the TPU belongs to a spanning network and 54% of it is trapped on average (range 40–59% over five realisations; Figure 4c). The generated morphology switches from droplet-in-matrix to co-continuous at 48 wt% TPU, following SEM observations [14].

Trapped TPU voxels are excluded from global co-degradation (Equation (14)) and are therefore subject only to hydrolysis (Equation (11)), the same slow pathway that governs neat TPU. Once PHBV and reachable TPU have been consumed, hydrolysis of the trapped TPU is the only active pathway.

Neat-PHBV kinetics. The 100/0 composition is the only one without a TPU phase. Two scale factors apply only to it: 0.90 multiplies *r*_collapse,PHBV_, and 0.04 multiplies the acceleration intensity A (effective A = 5.1). They were fitted to the neat-PHBV data (days 28–36) and have no independent physical origin. Figure 4 plots the composition-dependent functions of the model.

### 2.8. Transport of Biomass and Moisture

Two scalar fields are transported through the lattice in every Monte Carlo step: first moisture, then biomass. Both are advanced using a double buffer: at the start of the step, the field is copied to a read buffer, and every gradient is evaluated against that frozen copy. This means that the magnitude of the transfers does not depend on the order in which voxels are visited. The transfers themselves are accumulated in place, and the biomass pass reads the moisture field that has already been updated by the moisture pass. Therefore, the scheme is Jacobi in its gradients and sequential in its accumulation, rather than fully simultaneous.

Transport is downhill and stochastic. For each voxel *i* and each of its six face neighbours *j*, a transfer is attempted only if the neighbour contains a smaller amount of the field than the voxel, and is accepted with a fixed probability $p_{Y}$ (0.98 for moisture, 0.90 for biomass):

(19) $$\Delta Y_{\left(i\to j\right)}=\alpha_{diff}\cdot \left(Y_{i}^{\left(\left(t\right)\right)}-Y_{j}^{\left(\left(t\right)\right)}\right),\;if\;Y_{j}^{\left(\left(t\right)\right)}<Y_{i}^{\left(\left(t\right)\right)}\;and\;\xi <p_{Y}$$

where *Y* ∈ {h, X} denotes moisture or biomass, the superscript (*t*) indicates that both values are read from the frozen buffer, ξ is a uniform random draw, and $\alpha_{diff}$ = 0.12 for both fields. The transfer is applied as $Y_{i}\leftarrow Y_{i}-\Delta Y_{i\to j}$ and $Y_{j}\leftarrow Y_{j}+\Delta Y_{i\to j}$. Moisture is clipped to the range [0, 1] and biomass is floored at zero.

Biomass transport is subject to two additional restrictions. Firstly, it is only permitted into neighbouring voxels with a moisture content above 0.2. This ensures that biomass cannot invade dry interior material and must follow the advancing moisture front. Secondly, the transferred amount is attenuated by a cross-affinity factor when the two voxels differ in material identity. This factor is equal to 1.0 when transferring between voxels of the same material, or into removed voxels, and 0.10 when transferring across a PHBV/TPU interface. This represents the reduced ability of a PHBV-adapted population to colonise the TPU phase.

Biomass is not lost when a voxel is removed. Eighty per cent of the removed voxel’s biomass is redistributed among its occupied neighbours in proportion to their material weight: 1.0 for PHBV and 0.05 for TPU. This occurs provided the voxel held at least 0.001 units of biomass and at least one occupied neighbour is available. This inheritance process ensures that the microbial population remains attached to the receding erosion front, rather than being destroyed along with the material it has consumed. Removal also sets the moisture level of the vacated site to one, so that the growing void network acts as a moisture reservoir that draws the moisture front inwards.

At *t* = 0, the system is inoculated isotropically: every polymer voxel lying on one of the six external faces of the lattice receives an initial biomass of *X*_0_ and a moisture content of 0.9. All interior voxels start with a biomass and moisture content of zero.

### 2.9. Simulation Algorithm, Time Scheme and Calibration Protocol

Each Monte Carlo step (MCS) corresponds to a time interval of Δ*t* = 0.16 days (3.84 h), meaning that 256 steps cover the full 41-day ISO 20200 test period. Within each step, the following operations are executed in the order shown in Figure 2c: (1) the consumed PHBV fraction pcf and the enzyme level *E* are updated from the phase counts of the previous step; (2) the lattice is swept once and, for every voxel currently classified as surface, the Monod kinetics of Equations (1)–(5) are advanced, the degradation probability of Equations (6)–(15) is assembled and the collapse trial of Equations (16) and (17) is performed. If no collapse occurs, a degradation trial is performed against a uniform random number to decrement structural integrity or remove the voxel, (3) the moisture field is transported, and (4) the biomass field is transported. Phase counts and the disintegration percentage are recorded every five steps, and the full lattice state is written to disk on the eight experimental sampling days for morphological analysis. The scheme is a rule-based kinetic Monte Carlo method: the acceptance probability of each event is derived from kinetic rates (Equations (6)–(17)) and compared with a uniform random number. Unlike energy-based lattice methods such as the Metropolis or Q-Potts algorithms [34,41], no Hamiltonian is defined and no system energy is computed or minimised.

The traversal has two characteristics. Firstly, in the simulation of the composting environment, only surface voxels are updated. A voxel is classified as surface if it lies on a lattice face or if at least one of its six face neighbours is empty. This implements surface erosion as the dominant mechanism, which is consistent with observations that microorganisms erode polymer surfaces and spread progressively into the bulk [14]. This also means that the interior of the specimen remains inert until the front reaches it. Secondly, the sweep updates the lattice in place. This means that voxels removed earlier in a step modify the surface classification and neighbour counts seen by voxels visited later in the same step. The disintegration percentage reported by the model is the fraction of the initial polymer voxel population that has been removed. This is the lattice analogue of the gravimetric weight loss measured experimentally.

Calibration followed a hierarchical strategy in which each stage fixed parameters that were not revisited by later stages. First, the Monod parameters of the PHBV phase were fitted against the neat PHBV specimen (100/0), isolating them from every TPU-specific mechanism. The hydrolysis parameters were then fitted against neat TPU (0/100), governed solely by Equation (11). Once the pure components had been fixed, the co-degradation constant and the collapse acceleration ($r_{codeg}$, A and $a_{thr}$) were fitted against the blends that reach completion (95/5 to 60/40), the penetration depth *δ* against the 50/50 blend and, finally, the two neat-PHBV factors against the 100/0 specimen. The remaining adjusted parameters were set during the development of the model against all eight compositions and were kept unchanged during these stages; in total, 22 parameters were adjusted to the disintegration data (Table A1, column “Origin”). Mechanisms whose parameters were not identifiable according to the identifiability analysis were removed from the model. After each change, all eight compositions were re-simulated. Days 0, 6 and 13 fall within the lag phase, before the onset of measurable degradation, and are therefore excluded from the error. The agreement between simulation and experiment is quantified by the root-mean-square error:

(20) $$RMSE\;=\;\sqrt{\frac{1}{n}\sum_{i}{\left(D_{sim,i}\;-\;D_{exp,i}\right)}^{2}}$$

where *D* is the percentage disintegration and the sum runs over the n fitted points (days 20, 28, 34, 36 and 41 of each composition). Unless stated otherwise, *D*_sim_ is the mean of twenty replicates and the global RMSE uses all *n* = 40 points; the RMSE excluding two anomalous experimental points (95/5 on day 28 and 80/20 on day 20; *n* = 38) and the mean of the per-replicate RMSEs are also reported.

### 2.10. Reference Experimental Data

The calibration data originate from an experimental study conducted by Martínez-Abad et al. [14], in accordance with the ISO 20200 standard [46]. This study examined eight PHBV/TPU compositions (100/0, 95/5, 90/10, 80/20, 70/30, 60/40, 50/50, and 0/100, w/w). In that study, PHBV and TPU were melt-blended in a Brabender-type internal mixer and formed into thin films of approximately 200 µm by compression moulding. Their morphology, consisting of spherical TPU domains dispersed in the PHBV matrix, was characterised by SEM [14]. The size of these domains increases with TPU content (Table 1). Biodegradation tests were performed in a synthetic solid composting substrate in accordance with ISO 20200. The specimens were incubated at 58 ± 2 °C under aerobic thermophilic conditions for 41 days. Disintegration was quantified as weight loss relative to the initial mass on sampling days 0, 6, 13, 20, 28, 34, 36 and 41.

In that study, all compositions containing less than 50 wt% TPU, including the 60/40 blend, disintegrated completely by approximately day 36. In contrast, the 50/50 blend reached approximately 68% after 41 days without a clear plateau, and neat TPU reached approximately 8%. The experimental disintegration values used in this work were digitised from Figure 6 of Martínez-Abad et al. [14] using WebPlotDigitizer on the eight sampling days. 

### 2.11. Stochastic Robustness, Sensitivity Analysis and Virtual Temperature Experiments

Stochastic robustness was assessed by running twenty independent replicates of each composition on the full 100 × 100 × 20 lattice. Each replicate used an independent seed to govern both RSA and co-continuous morphology generation, as well as the Monte Carlo acceptance trials. The reported variability therefore includes both morphological and kinetic stochasticity. The RMSE of each replicate was computed against the experimental reference, and the mean, standard deviation, coefficient of variation, and range across replicates are reported for each composition and overall.

The Morris elementary-effects method [47] was used to rank the influence of the parameters and to test for over-parameterisation, as implemented in SALib [48]. This global screening technique, which varies one parameter at a time, computes elementary effects along trajectories in parameter space. It provides two statistics per parameter: the mean of the absolute effects µ*, which measures overall influence [49], and their standard deviation σ, which measures non-linearity and interaction [47]. This method is efficient enough to be used with computationally costly models, for which variance-based Sobol indices [50] would be impractical. Its trajectories were spatially optimised following Campolongo et al. [49]. Twelve parameters spanning all model components were varied within ±50% of their calibrated values: *µ*_max,PHBV_, *K*_S,PHBV_, *µ*_max,TPU_, *p*_hyd_, *r*_collapse,PHBV_, $r_{codeg}$, *p*, *λ*_E_, *A*, $a_{thr}$, the neat-PHBV base factor and *δ* (integer values 1–3). The PHBV collapse rate and *λ*_E_ were capped at unity, since larger values are not physically meaningful; as elementary effects are computed on the normalised unit hypercube, µ* for these two parameters is a conservative estimate. All simulations of the design used the same random seed, so that elementary effects are not contaminated by replicate-to-replicate noise. The design generated 130 samples per composition on a reduced 50 × 50 × 10 lattice using the disintegration percentage at day 28 as the response metric (*r* = 10 trajectories spatially optimised from a pool of 50, 6 levels). The analysis was carried out for four compositions (100/0, 80/20, 50/50 and 0/100) chosen to span the three degradation regimes.

The thermal response of the model was examined through virtual experiments conducted at ten temperatures spanning the cardinal range (15 °C, 20 °C, 25 °C, 30 °C, 37 °C, 45 °C, 52 °C, 58 °C, 64 °C and 68 °C). Only the environmental temperature was altered, with the change propagating into the model exclusively through *f*(*T*) in Equation (1). No other parameters were adjusted. Each simulation was run until *t*_50_ was reached. At 15 °C, this required 730 simulated days. The half-life (*t*_50_) of neat PHBV and the disintegration of neat TPU on day 41 were recorded at each temperature: the former to verify the expected 1/*f*(*T*) scaling of the biotic pathway, and the latter to verify that the abiotic pathway is decoupled from *f*(*T*), as defined in Equation (11).

### 2.12. Predictive Validation, Model Comparison and Identifiability Analysis

To separate goodness of fit from predictive ability, a hold-out test was performed. The calibration steps that involve the blends ($r_{codeg}$, then $a_{thr}$, with *A* kept at its calibrated value) were repeated using only the 95/5, 80/20, 60/40 and 50/50 blends, and the intermediate 90/10 and 70/30 blends were withheld. With all parameters fixed, the withheld blends were simulated (twenty replicates each) and compared with the experimental data.

The hold-out test was complemented by a leave-one-composition-out cross-validation over the six blends. For each blend, the blend-level calibration step (a joint grid over *r*_codeg_ ∈ {1.2, 1.5, 1.8, 2.1, 2.4, 2.7} and *a_thr_* ∈ {0.40, 0.45, 0.50}, three replicates) was repeated on the other five blends, and the withheld blend was then predicted with twenty replicates; all other parameters were kept at their final values. Applied to all six blends, the same grid returns the final values (*r*_codeg_ = 1.8, *a_thr_* = 0.45).

The contribution of the model components was assessed by comparing nested versions of the model: M0, without the percolation regime (a hard threshold at *w*_PHBV_ = 0.55 that excludes the 50/50 blend from co-degradation) and without the neat-PHBV factors; M1, M0 plus the topological percolation regime; and M2, the final model. Two empirical references were also fitted by least squares to all experimental points (days 0–41): a modified Gompertz function fitted independently to each composition (three parameters per composition, 24 in total) and a logistic function whose plateau, midpoint and slope depend on *w*_TPU_ (six parameters in total). All models were compared through the RMSE over the same 40 points and through the prediction RMSE of the hold-out test.

Practical identifiability was assessed in two ways. First, one-dimensional RMSE profiles were computed by setting each profiled parameter to 0.5, 0.75, 1.25 and 1.5 times its final value, with all other parameters fixed (three replicates per point with common random seeds). Second, the collinearity index *γ* of Brun et al. [51] was computed from the local sensitivity matrix of the 40 fitted outputs with respect to the logarithm of each parameter, obtained by central differences (×0.75 and ×1.25); values of γ above 10–15 indicate that the parameter set cannot be identified from the data. This analysis was first applied to an earlier version of the model that contained additional mechanisms.

## 3. Results

### 3.1. Model Calibration and Degradation Regimes

**Figure 5:**
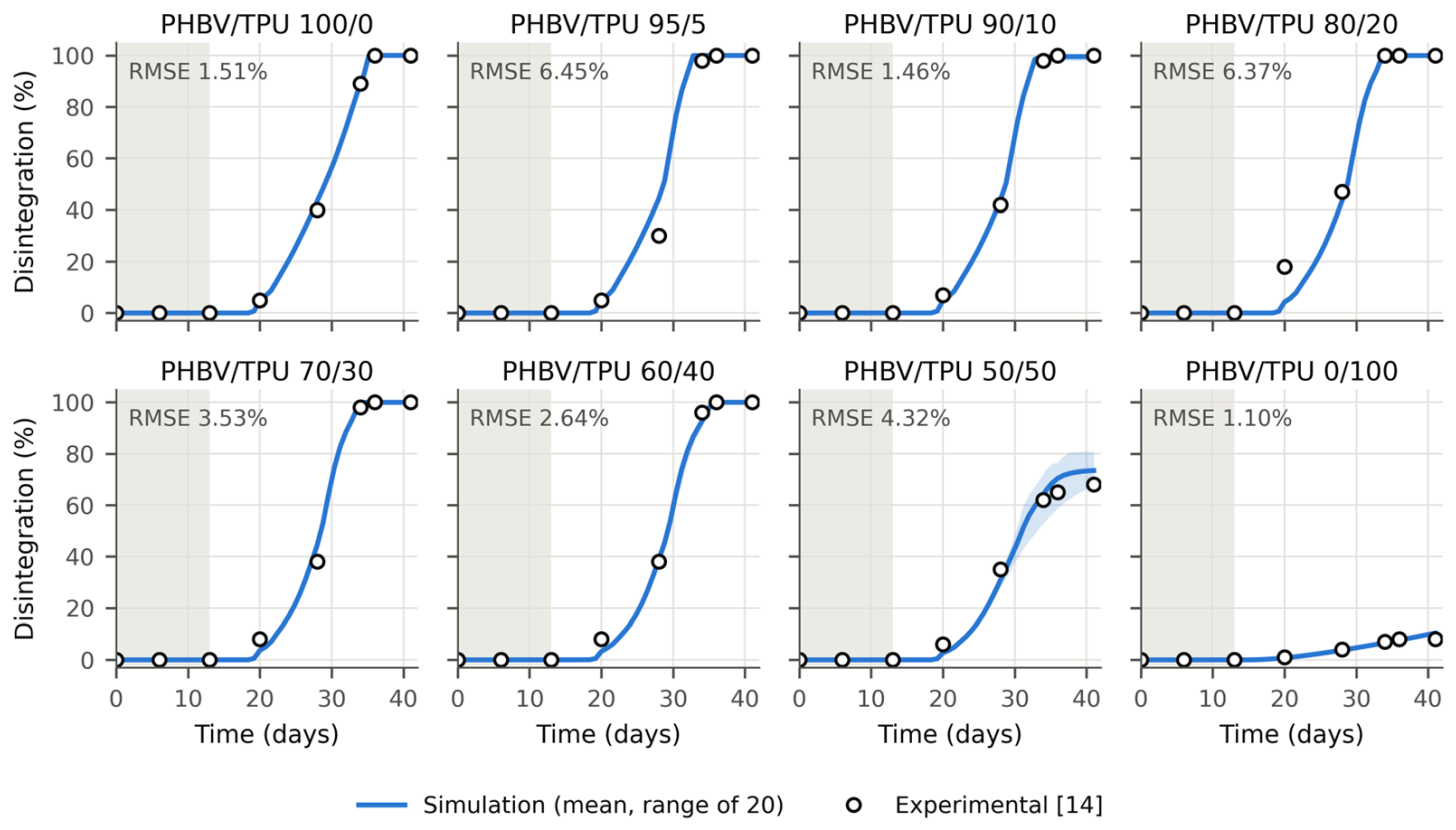
Disintegration curves by composition: simulation (line, mean of 20 replicates; shaded band, range of the replicates) versus experimental data (markers, Martínez-Abad et al. [14]) for the eight PHBV/TPU compositions under ISO 20200 at 58 °C over 41 days. The RMSE of each panel is computed on days 20–41; the grey band marks the lag phase (days 0–13), which is excluded from the RMSE.

Figure 5 compares the simulated and experimental disintegration curves of the eight compositions over the 41-day test period. The lowest RMSEs were obtained for neat TPU (0/100, 1.10%), the 90/10 blend (1.46%) and neat PHBV (1.51%). The 50/50 blend had an RMSE of 4.32%, with 73.5% simulated versus 68% experimental disintegration on day 41.

Table 2 lists the simulated and experimental values for the five fitted sampling days, together with the per-composition RMSE.

**Figure 6:**
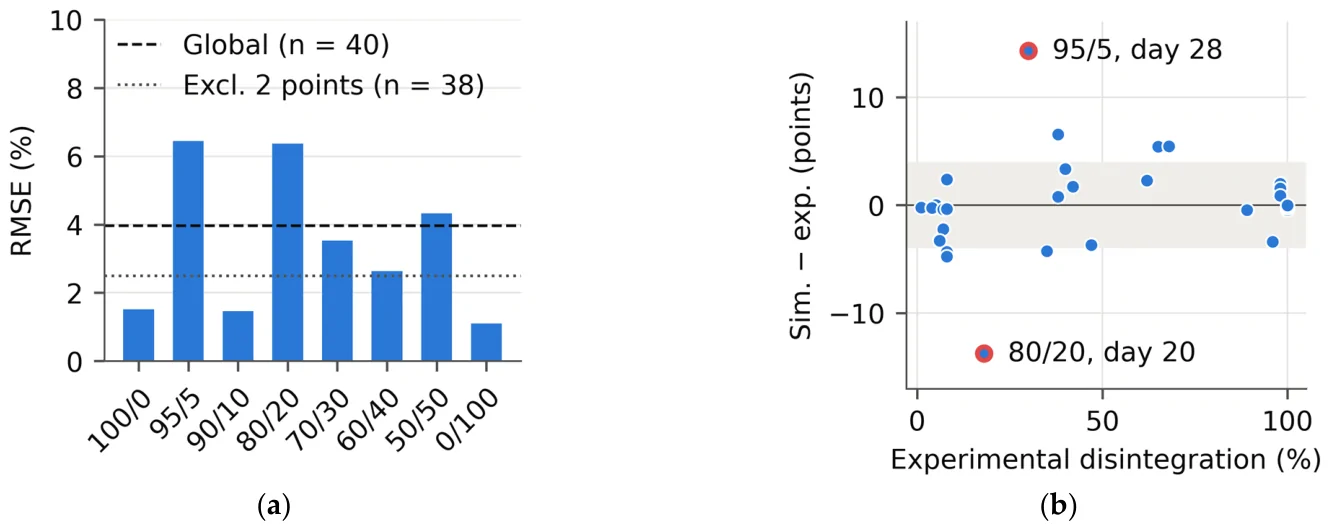
(**a**) RMSE by composition, with the global RMSE (3.97%, n = 40) and the RMSE excluding the two anomalous points (2.49%, n = 38). (**b**) Residuals (simulation minus experiment) versus experimental disintegration; the grey band marks ±4 points. The two anomalous points (95/5, day 28; 80/20, day 20) are outlined in red.

The global RMSE (Equation (20)) over all 40 fitted points was 3.97% (Figure 6a); this is the primary measure of agreement used in this work. Two experimental points break the monotonic dependence on composition shown by all other data of the complete-disintegration regime: the 95/5 blend on day 28 (30%, compared with 40% and 42% for the neighbouring 100/0 and 90/10 compositions) and the 80/20 blend on day 20 (18%, compared with 7% and 8% for 90/10 and 70/30). A model-independent criterion—a deviation of more than 10 percentage points from the mean of the two neighbouring compositions on the same day—identifies these two points and no others (the next largest deviation is 7 points). Because they cannot be confirmed as experimental artefacts from the published data [14], they are retained in the primary metric, and the RMSE with them excluded (2.49%, *n* = 38) is reported only as a sensitivity figure. Figure 6b shows that the remaining residual error is concentrated in the day-28 value of the 70/30 blend (+6.5 points) and the last two values of the 50/50 blend (+5.4 and +5.5 points), and that day-20 disintegration is underestimated by 2–5 points in the 90/10, 70/30, 60/40 and 50/50 blends.

### 3.2. Morphological Evolution of Degradation

**Figure 7:**
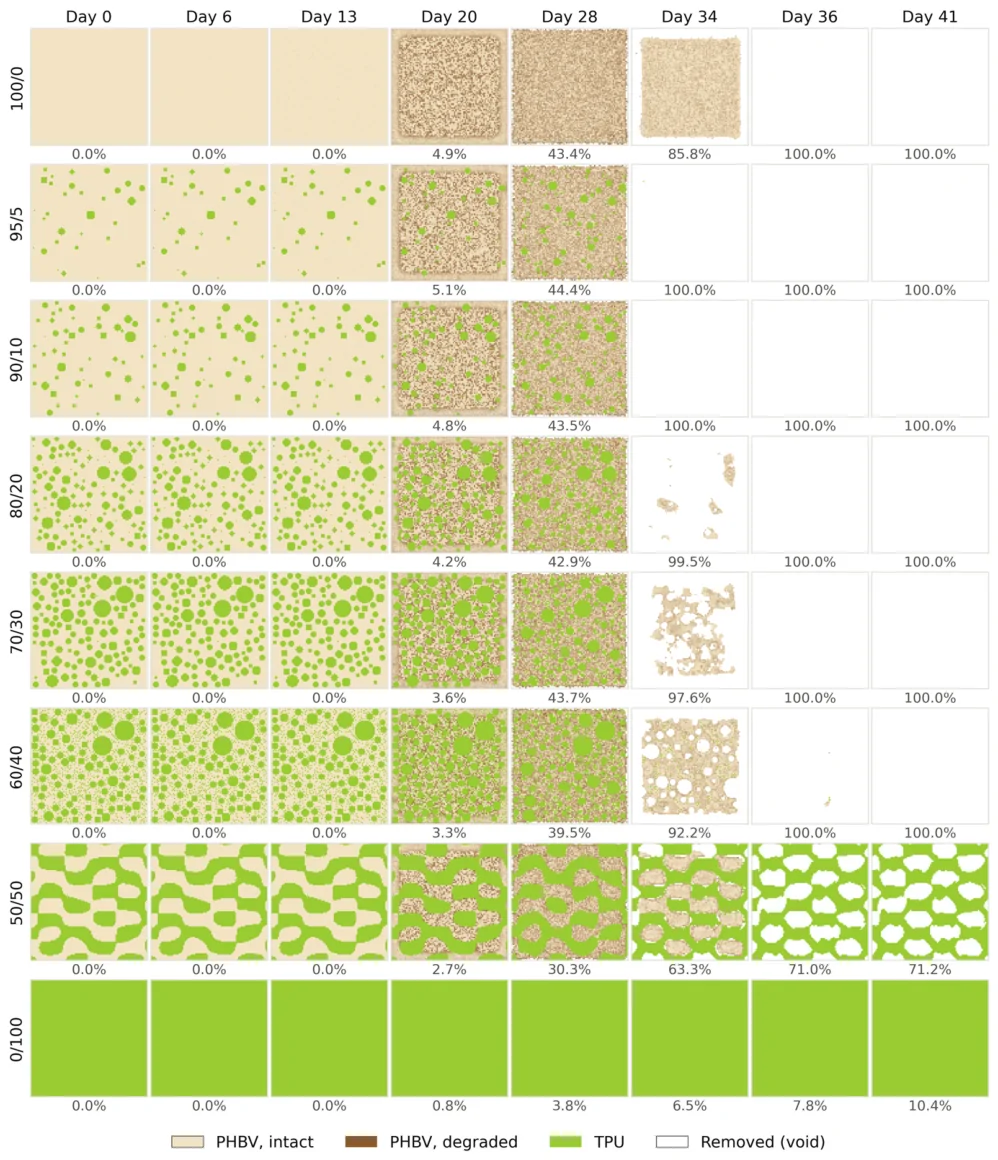
Morphological evolution (top view) for the eight compositions (rows) on the eight sampling days (columns); single realisation (seed 1). PHBV is shown from cream (intact) to brown (degraded) according to its structural integrity; TPU is yellow-green; removed voxels are white. Each panel is labelled with the disintegration of the realisation. In the 50/50 blend, the trapped TPU network persists after 41 days. The isolated TPU voxels visible in the 60/40 blend are the residual filling placed after RSA saturation. Domain: 100 × 100 × 20 voxels.

The model’s three-dimensional spatial resolution allows the morphological evolution of degradation to be visualised throughout the test (Figure 7). The top-view projections on the sampling days differ between compositions.

In PHBV-rich blends (100/0 to 60/40), the matrix remains practically intact during the lag phase (days 0–13). From day 20 onwards, edge erosion and the formation of voids begin. By day 28, fragmentation has become widespread, and the material has disappeared completely by days 34–36.

In the 50/50 transition blend, the PHBV phase is completely eroded, but the TPU domains—visible as a persistent yellow-green network—remain after 41 days. The voxel count for this realisation shows that all the PHBV has been consumed, while more than half of the TPU remains (about 57,600 of 100,000 voxels). This corresponds to 71.2% disintegration for this particular realisation, compared with the replicate mean of 73.5% (Table 2).

In neat TPU (0/100), the domain thins slowly and uniformly without fragmenting, and disintegration remains at approximately 10%.

### 3.3. The 50/50 Percolation Transition

**Figure 8:**
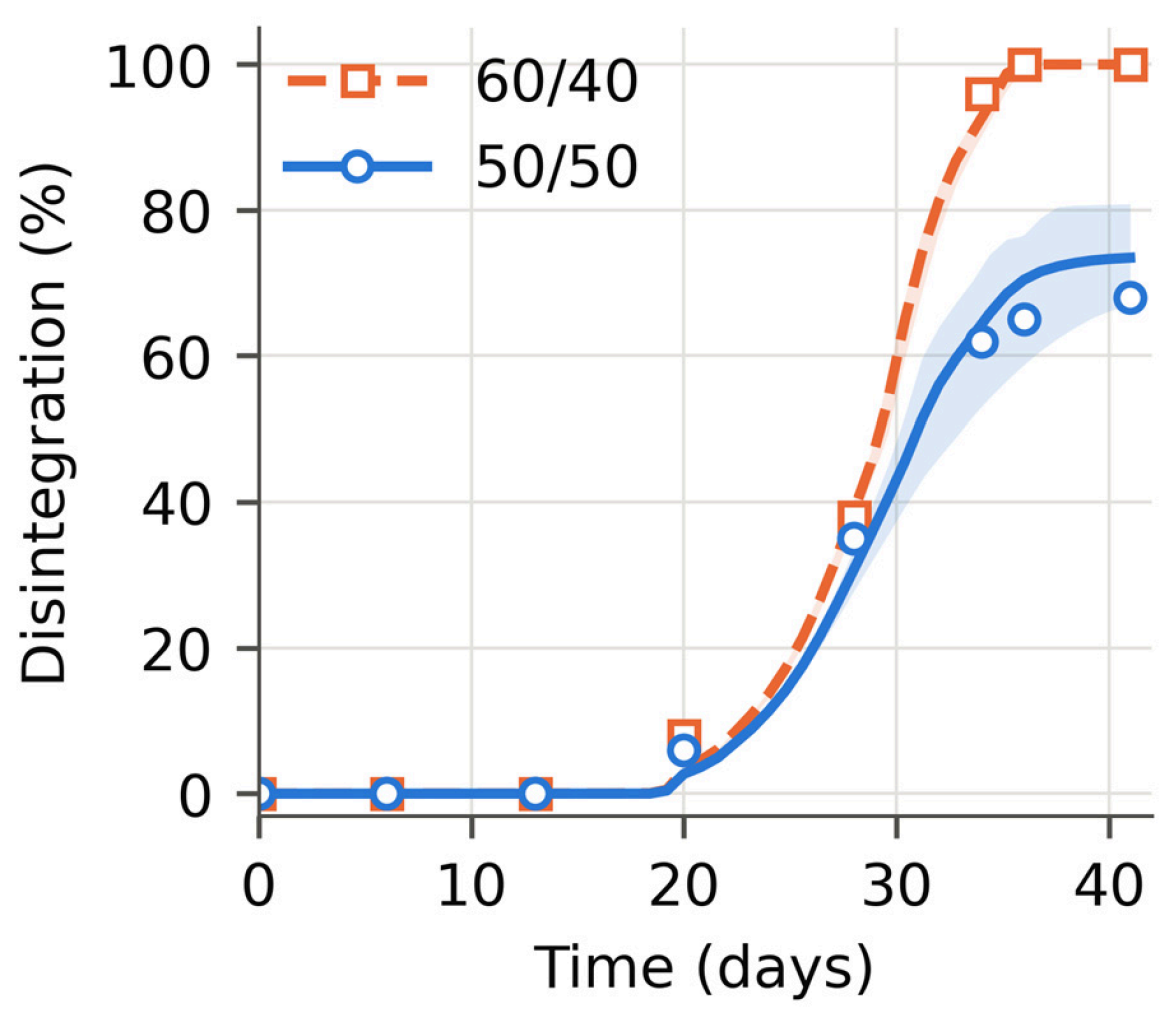
The 50/50 transition blend (incomplete disintegration; solid line) compared with the 60/40 blend, which completes (dashed line). Lines: mean of 20 replicates; shaded bands: range of the replicates; markers: experimental data [14].

The simulated 50/50 blend does not reach complete disintegration (Figure 8). The reachable TPU (46% of the TPU on average) co-degrades as the PHBV matrix is consumed, increasing disintegration from ~31% on day 28 to ~64% on day 34. Thereafter, depletion of the matrix stops co-degradation; the trapped TPU is subject only to hydrolysis, and disintegration increases more slowly, reaching ~74% on day 41.

The simulated 50/50 curve starts at a rate comparable to that of the PHBV-rich blends and does not reach completion. Martínez-Abad et al. [14] describe the curve as showing ‘no tendency to plateau’; the data extracted from their Figure 6 show a deceleration but no flat plateau (62/65/68% at days 34/36/41). The simulated curve rises from 64% to 74% between days 34 and 41, overestimating day 41 by 5.5 points (RMSE 4.32%).

### 3.4. Stochastic Robustness

Stochastic variability was assessed with twenty replicates per composition, each with a different seed. The global RMSE was 4.16% ± 0.28% (mean of the replicates), with a coefficient of variation of 6.7% and a range of 3.86–4.78% (Table 3).

Variability is markedly composition-dependent. It is lowest for the 80/20 and 95/5 blends, neat TPU and neat PHBV (CV of 0.9–2.0%), intermediate for the 90/10, 70/30 and 60/40 blends (CV of 5.3–10.1%), and highest for the 50/50 blend (CV of 30%); in this blend, the trapped fraction ranged from 40% to 59% of the TPU across five generated morphologies (Figure 4c).

The RMSE of the replicate-averaged curve used in Table 2 (3.97%) is 0.19 points lower than the mean per-replicate RMSE (4.16%). According to Jensen’s inequality, the RMSE of the average curve cannot exceed the quadratic mean of the per-replicate RMSEs; this bound was verified and is satisfied for all eight compositions.

### 3.5. Morris Sensitivity Analysis

**Figure 9:**
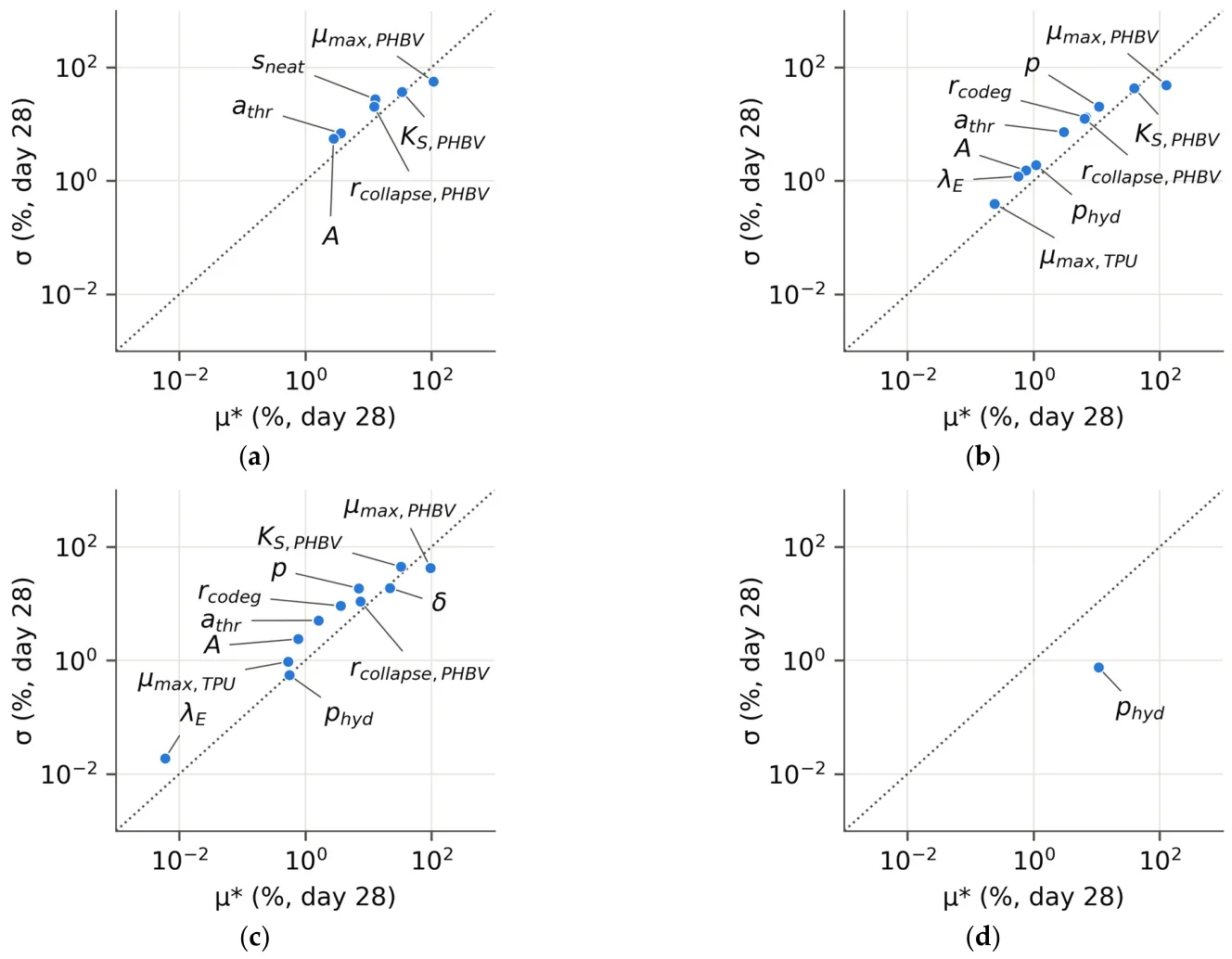
Morris sensitivity analysis (µ* versus *σ*, logarithmic axes) of twelve model parameters for four compositions: (**a**) neat PHBV; (**b**) 80/20; (**c**) 50/50; (**d**) neat TPU. Response: disintegration at day 28. Design: ten spatially optimised trajectories selected from a pool of fifty, six levels, 130 simulations per composition, 50 × 50 × 10 domain, and fixed random seed. Parameters with µ* = 0 are not shown; the dotted line marks *σ* = µ*.

Figure 9 shows the Morris sensitivity measures for the four compositions. For neat PHBV (100/0), Monod kinetics dominate (*µ*_max,PHBV_, µ* = 107.7; *K*_S,PHBV_, µ* = 34.1), followed by the neat-PHBV base factor (µ* = 12.8), the PHBV collapse rate (µ* = 12.3), $a_{thr}$ (µ* = 3.6) and *A* (µ* = 2.8); all TPU-specific parameters have µ* = 0. In the 80/20 blend, Monod kinetics still dominate (µ* = 127.2 and 39.5), followed by the co-degradation exponent *p* (µ* = 10.9), $r_{codeg}$ (µ* = 6.7) and the PHBV collapse rate (µ* = 6.5), whereas *δ* has µ* = 0. In the 50/50 blend, *δ* becomes the third most influential parameter (µ* = 21.9), after the Monod constants (µ* = 96.1 and 32.9), followed by the PHBV collapse rate (µ* = 7.5), *p* (µ* = 7.0) and $r_{codeg}$ (µ* = 3.6). For neat TPU (0/100), the hydrolysis probability is the only influential parameter (µ* = 10.8), with *σ* = 0.8.

The three most influential parameters in the PHBV-containing compositions show *σ*/µ* ratios between 0.4 and 2.2 (0.07 for the hydrolysis probability in neat TPU), whereas most parameters lie in the lower-left region of the µ*–*σ* diagram.

### 3.6. Virtual Temperature Experiments

**Figure 10:**
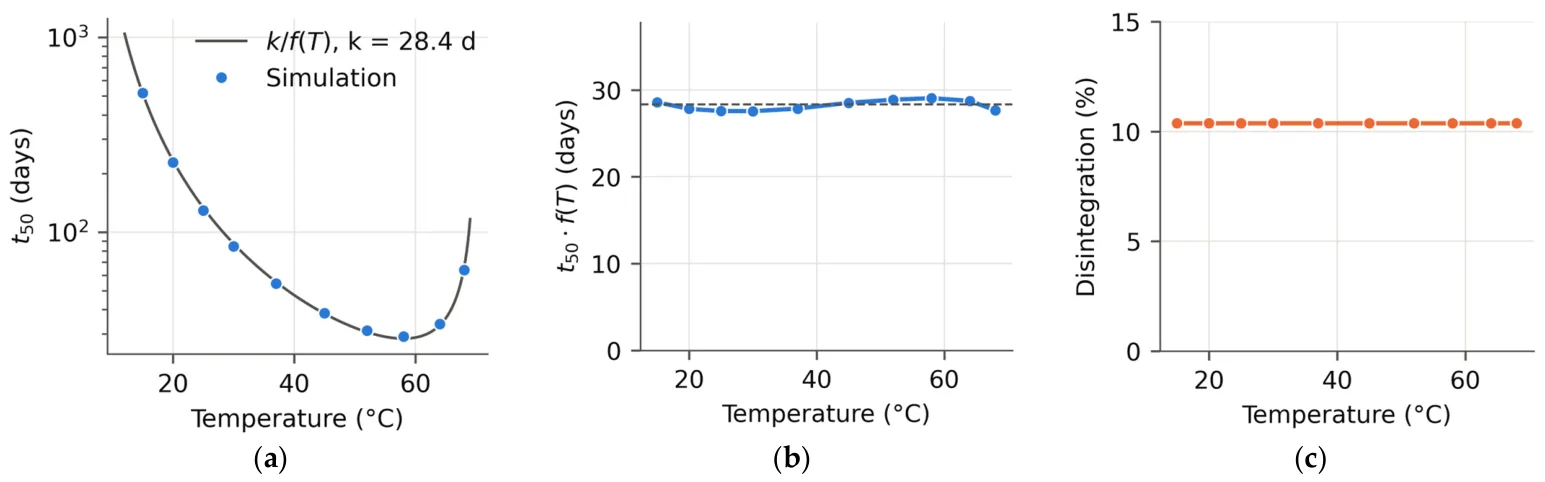
Virtual temperature experiments (15–68 °C). (**a**) Half-life *t*_50_ of neat PHBV versus temperature (mean of three replicates); the line is the least-squares fit *t*_50_ = *k*/*f*(*T*), *k* = 28.4 days (*R*^2^ = 0.9997). (**b**) Product *t*_50_·*f*(*T*) (mean 28.2 days, CV 2.0%). (**c**) Disintegration of neat TPU at day 41, which is 10.4% at every temperature because Equation (11) does not depend on temperature by construction.

Figure 10 shows the results of the virtual temperature experiments, in which no parameter was re-tuned.

The half-life of neat PHBV scales as 1/*f*(*T*) across the entire cardinal range. Within the 15–68 °C range, *t*_50_ varies by a factor of 17.8, ranging from 29.1 days at the thermal optimum of 58 °C to 518.6 days at 15 °C. The product *t*_50_·*f*(*T*) remains constant at 28.23 days (CV 2.0%), including 68 °C, above the optimum (*f*(*T*) = 0.43). Fitting *t*_50_ = *k*/*f*(*T*), with *k* = 28.38 days, yields *R*^2^ = 0.9997, with a maximum relative error of 2.9%.

At day 41, the disintegration of neat TPU is 10.4% at every temperature between 15 and 68 °C (Figure 10c).

### 3.7. Hold-Out Test, Model Comparison and Identifiability

The identifiability analysis was first applied to an earlier version of the model that contained four additional mechanisms: substrate-depletion collapse of TPU, local co-metabolisation of TPU adjacent to eroded PHBV, a late exposure boost of TPU once the matrix was nearly consumed, and a surface retardation of PHBV collapse in blends; it also included a scaling of co-degradation by the PHBV/TPU ratio and an activation threshold that decreased linearly with TPU content. For the first four, and for the slope of the threshold ramp, the RMSE changed by less than 0.07 points over ±50% of their calibrated values, and the optimum of the ratio scaling made it inactive (a value of one for all blends). These mechanisms were removed and the remaining parameters were recalibrated. The resulting model (Table A1) reaches the same global RMSE as the earlier version (3.97%) with 22 instead of 35 adjusted parameters.

**Figure A1:**
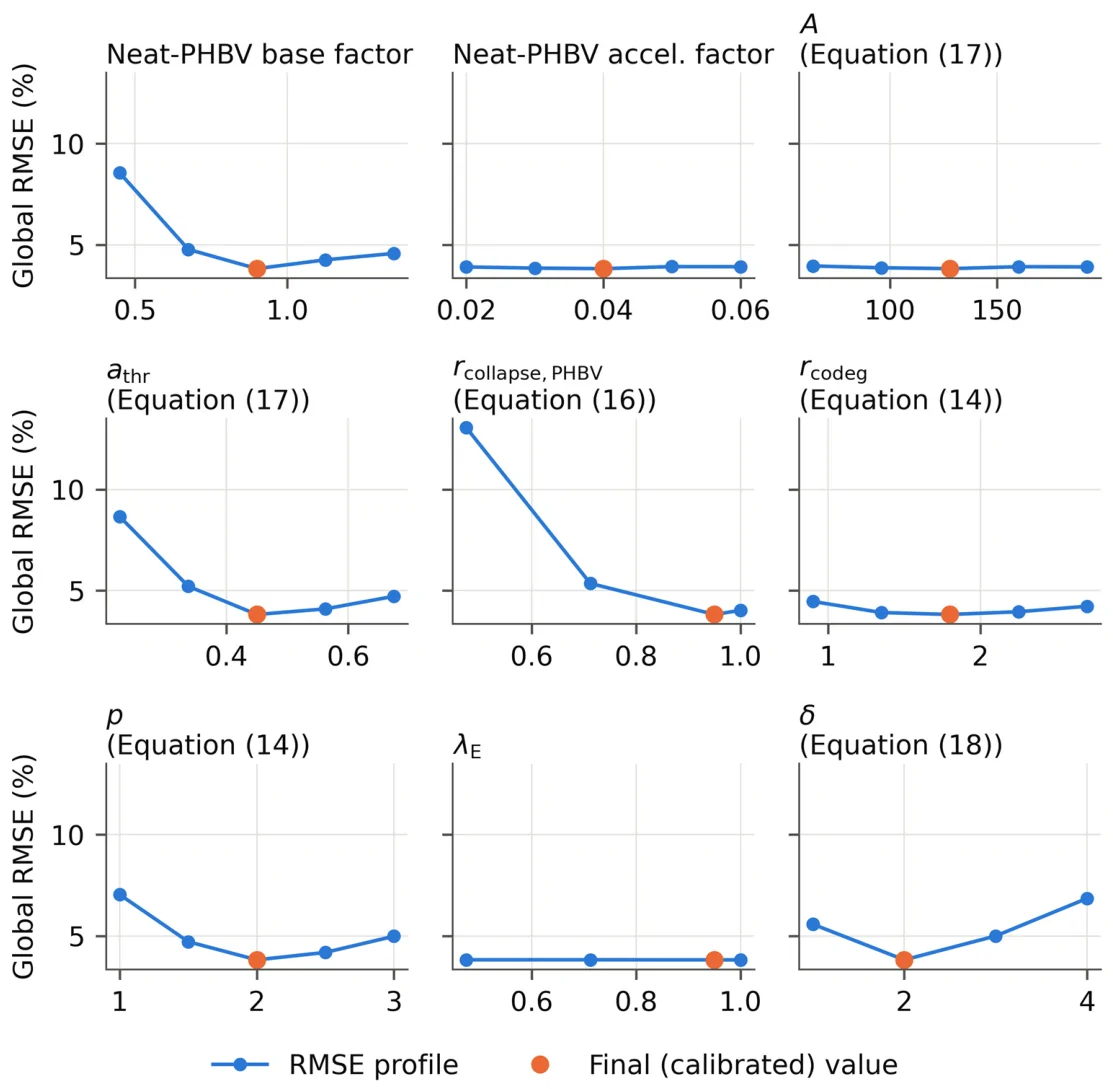
One-dimensional RMSE profiles of nine parameters of the final model (seven calibrated and two fixed a priori, *p* and *λ*_E_) ((global RMSE over the 40 fitted points, mean of three replicates with common random seeds). Each parameter was set to 0.5, 0.75, 1.25 and 1.5 times its final value (orange marker) with all others fixed, and integer values 1–4 were used for *δ*. Blue line, RMSE profile. Orange dot, final (calibrated) value. The flat profile of *λ*_E_ shows that it cannot be identified from the present data.

In the final model, all RMSE profiles show a minimum at the calibrated value except that of *λ*_E_, which is flat (Figure A1). The profile of *A* is shallow above about 100. The collinearity index of the nine profiled parameters is *γ* = 34.8; the most collinear pair is $r_{codeg}$ and *p* (*γ* = 10.2). With *λ*_E_ and *p* fixed a priori, the index falls to *γ* = 6.6, below the threshold of 10–15.

When the 90/10 and 70/30 blends were withheld, the recalibration on the remaining blends selected $r_{codeg}$ = 2.1 (instead of 1.8) and the same $a_{thr}$. The blind predictions for the withheld blends gave RMSEs of 1.59% (90/10) and 4.29% (70/30), or 3.23% combined, compared with 2.70% (1.46% and 3.53%) when these blends were included in the calibration. The largest error is the day-28 value of the 70/30 blend, which is also overestimated in the reference calibration. The prediction error is lower than that of the composition-parametric logistic function under the same test (3.68%) and slightly higher than that of the earlier, more complex version of the model (2.90%).

In the leave-one-composition-out cross-validation, *a_thr_* = 0.45 was selected in all six folds and *r*_codeg_ varied between 1.5 and 2.1. The prediction RMSE of each blend was 6.45% (95/5), 1.46% (90/10), 6.54% (80/20), 4.29% (70/30), 3.08% (60/40) and 4.46% (50/50), against fitted values of 6.45%, 1.46%, 6.37%, 3.53%, 2.64% and 4.32%. The pooled prediction RMSE over the 30 blend points was 4.73%, compared with 4.52% for the same points in the full calibration (3.15% and 2.79%, respectively, excluding the two anomalous points). For the 50/50 blend, this prediction retains *δ* = 2 voxels, which is informed only by that blend; with *δ* = 1 or 3 voxels, the prediction RMSE rises to 10.6% and 12.1%, respectively.

Table 4 compares the nested versions of the model and the empirical references. Removing the neat-PHBV factors (M1) increased the global RMSE from 3.97% to 4.37%, entirely through neat PHBV (5.39%), which then does not complete disintegration within the test. Removing the percolation regime as well (M0) increased it to 8.27%, almost entirely through the 50/50 blend (20.3%), which then remains below 50%. Neither component affects the prediction of the withheld blends, which contain no trapped TPU. The per-composition Gompertz fit reached an RMSE of 4.61% with 24 parameters, and the six-parameter logistic function an RMSE of 5.26%. The lattice model has more adjustable parameters than the logistic function, and the Akaike information criterion (AIC) [52], which penalises the number of parameters, favours the six-parameter logistic function for these 40 points.

## 4. Discussion

### 4.1. Composition-Dependent Degradation Regimes

Three degradation regimes can be identified. In blends with up to 40 wt% TPU, the PHBV phase degrades rapidly after the lag phase through Monod-coupled erosion. In the model, the TPU phase is then removed by enzymatic co-degradation (Equation (14)) [12,53], with accessibility factors (Equation (15)) ranging from 0.38 (95/5) to 1.0 (60/40). In the droplet-in-matrix blends, which contain no spanning TPU network, all of the TPU is reachable, and all of these compositions, including the 60/40 blend, disintegrate completely within the 41-day test. The second regime is the 50/50 transition blend, where the PHBV phase degrades but only the reachable TPU fraction co-degrades. The trapped fraction is subject to slow abiotic hydrolysis and persists, limiting disintegration to ~74% (68% experimentally) with no flat plateau. The third regime is neat TPU, which degrades minimally (~10% at 41 days), with degradation governed by the abiotic hydrolysis of the soft segment [12,45]. This degradation profile is linear, in contrast to the sigmoidal profiles of the blends; in the model, this difference results from the absence of the positive biomass–substrate–degradation feedback loop.

In the model, the boundary between complete and incomplete disintegration lies between 40 and 50 wt% TPU because the generated morphology changes from droplet-in-matrix to co-continuous in that range, as observed by SEM [14], and only the co-continuous morphology contains a spanning TPU network; the location of the boundary is therefore inherited from the morphology rather than predicted. Within this framework, the model shows that a reachable/trapped partition of the TPU phase, combined with the exclusion of the trapped fraction from co-degradation, is sufficient to reproduce the shape of the incomplete 50/50 curve without a discontinuous switch in the kinetics. This interpretation is consistent with the observation that the 50/50 blend exhibits only slight surface roughening and is still present after 41 days [14], unlike the complete fragmentation observed at lower TPU contents, but it does not demonstrate that percolation is the physical cause of the incomplete disintegration.

To make explicit which results are imposed and which arise from the simulation, Table 5 separates the behaviours prescribed by model rules or fitted parameters from those that emerge from the interaction of the mechanisms. In particular, the location of the boundary between complete and incomplete disintegration is inherited from the morphology generator, which switches to a co-continuous structure at 48 wt% TPU following SEM observations, and the size of the trapped fraction in the 50/50 blend depends on the calibrated penetration depth *δ*. What the simulation adds is how this fraction arises from the generated network and the timing and shape with which the final incomplete level is approached.

### 4.2. Role of the RSA Morphology

The RSA algorithm produces discrete spherical domains with a log-normal size distribution calibrated against SEM data, capturing the growth of the mean domain size with TPU fraction (2.0 voxels at 5%, increasing to 5.0 at 40%), which is consistent with the observed droplet coalescence and transition to a co-continuous morphology above the percolation threshold reported for the 50/50 blend [14]. In the model, morphology influences the results mainly through the surface-to-volume ratio of the TPU domains, which decreases with mean radius. Small, isolated domains in low-TPU blends expose a proportionally greater interface area than larger, co-continuous structures found at higher TPU content. This effect is captured at two scales: the local per-voxel exposure to the eroding matrix (Equation (12)) and the global enzymatic accessibility (Equation (15)), which acts as a composition-level proxy for the same geometric trend. The connectivity of the PHBV phase is audited using a breadth-first search, which confirms the percolation of the matrix in every blend that admits a sea-island morphology. This audit is diagnostic and does not determine the outcome.

### 4.3. Enzymatic Co-Degradation and Collapse Acceleration

The co-degradation mechanism offers a possible explanation for the complete disintegration of TPU in PHBV-rich blends, as opposed to neat TPU, which disintegrates by only ~10%. The model assumes that PHBV consumption generates increasing reservoirs of extracellular esterases and lipases, produced by microorganisms that attack the ester bonds of PHBV (poly(3-hydroxybutyrate) depolymerases and related hydrolases [33,53,54]), which may also attack the ester bonds of TPU. Such cross-reactivity has been reported for several polyester structures [12,45,54]. The sigmoidal shape of the blend curves is reproduced by a single acceleration of PHBV collapse that becomes active once 45% of the polymer has been removed, which can be interpreted as the increase in exposed surface area as the film fragments into ever smaller pieces [14]. The simulated erosion sequence (voids from day 20, widespread fragmentation by day 28 and complete loss by days 34–36) follows the reported progression of visible fractures from day 20, breakage into small pieces by day 28 and loss of visible fragments by day 36 [14]. A single threshold, common to all blends, is sufficient: the composition-dependent threshold of an earlier version of the model was not supported by the data in the identifiability analysis. Neat PHBV requires a weaker acceleration and a slightly lower base rate, which is consistent with the absence of TPU domains whose interfaces promote fragmentation in the blends, although this interpretation remains to be tested experimentally.

The persistence of the TPU phase where co-degradation is incomplete is not specific to the compression-moulded films modelled here. Under ISO 14855-1 [55] mineralisation testing of injection-moulded PHBV/TPU compounds, neat PHBV exceeded 90% conversion while the blends plateaued near 75%, with at least 5% of the TPU mineralised over 90 days [13]. The metric and specimen geometry differ from those modelled in this work, but the qualitative persistence of the elastomeric phase is consistent with the trapped-fraction mechanism proposed here.

### 4.4. Sensitivity Hierarchy, Identifiability and Model Complexity

The Morris analysis [47,48] shows that, in each composition regime, only a few parameters govern the outcome and that these correspond to the mechanisms active in that regime: Monod kinetics in the PHBV-rich blends, the penetration depth of the percolation regime in the 50/50 blend and hydrolysis in neat TPU. The *σ*/µ* ratios of the three most influential parameters in the PHBV-containing compositions (0.4–2.2) indicate non-linear effects and interactions [47], consistent with the coupling between biomass growth, substrate consumption and degradation. This consistency is expected by construction and does not by itself exclude overfitting, which was therefore examined separately. The identifiability analysis led to the removal of four mechanisms and two composition-dependent functions that were not supported by the data, reducing the number of adjusted parameters from 35 to 22 without loss of fit, and showed that the remaining set is identifiable once *λ*_E_ and *p* are fixed. The RMSE profile of *λ*_E_ is flat because enzyme decay only acts once the matrix is exhausted, the profile of A is shallow above about 100 because the removal probability saturates, and $r_{codeg}$ and *p* are collinear because both control the rate at which the reachable TPU is removed as the matrix is consumed. The comparison with simpler models (Table 4) shows that the percolation regime and the neat-PHBV factors are each needed to reproduce the data, and that the model predicts withheld compositions more accurately than an empirical logistic description. In the leave-one-composition-out cross-validation, the prediction error of the blends increases only slightly relative to the fitted error (4.73% versus 4.52%), and the blend-level parameters remain stable across folds, which argues against an over-fit to individual compositions. The final level of the 50/50 blend, however, depends on *δ*, which can only be constrained by that blend; it is therefore fitted rather than predicted. Information criteria such as the AIC favour the six-parameter logistic function, but they assume independent residuals and only measure the ability to reproduce the measured curves; the lattice model additionally provides spatial and mechanistic information (Figure 7 and Figure 8). The number of parameters nevertheless remains large relative to the 40 available data points, and testing the model against independent experimental series is the most direct way to strengthen it.

### 4.5. Thermal Behaviour Implied by the Model

The invariance of the product *t*_50_·*f*(*T*) across the 15–68 °C interval, including the supra-optimal regime, shows that the CTMI correction [44] is consistently integrated into the growth kinetics, so that the thermal response of the biotic pathway follows the cardinal-temperature model without numerical artefacts. The constant disintegration of neat TPU at all temperatures is a direct consequence of the model definition, in which Equation (11) does not depend on *f*(*T*); it confirms that the two pathways are decoupled in the code, but it is not evidence that TPU hydrolysis is temperature-independent. Both results are therefore verifications of the implementation rather than a validation: the model has been calibrated at a single temperature (58 °C), and its thermal predictions rest on the assumption that the cardinal temperatures and the CTMI shape describe the composting consortium, which has not been tested experimentally for these blends. Since the hydrolysis of polyurethane ester segments is thermally activated [45], an Arrhenius-type dependence of Equation (11) will be required before the model is used away from 58 °C. Disintegration tests of the same blends at two or more additional temperatures are needed to validate both pathways; until then, the virtual experiments should be regarded as illustrating the consequences of the model assumptions for thermal profiles in industrial composting [56], not as predictions.

### 4.6. Comparison with the Literature

The present model can be placed in the context of previous approaches to modelling polymer biodegradation. While first-order-with-lag kinetic models [27] can reproduce individual mineralisation curves, they capture neither composition dependence nor spatial structure. Continuum reaction–diffusion models [24,25] and their extensions incorporating chain scission, crystallinity, Avrami coupling [30,31,32], improved chain-cut statistics [37] and multiscale oligomer transport [38] describe the degradation of a single material in detail, typically neat PLA or PHAs. However, they do not address heterogeneous blends with a recalcitrant phase. Classical microbial growth kinetics [43] have been extended to degradation through Monod-type models of anaerobic digestion [26]. The differential-equation systems for PLA [28] or starch–polyolefin blends [29] incorporate microbial activity, but in lumped-parameter (0-D) formulations that do not resolve morphology. Monte Carlo models, an approach with roots in the original stochastic method [34], have provided spatial resolution. The lattice erosion model of Göpferich and Langer [23] reproduced erosion fronts, porosity evolution and microstructural change by assigning stochastic lifetimes to matrix pixels that erode only after contact with an eroded neighbour. Three-dimensional implementations include the poly(lactide) scaffold degradation of Mohammadi and Jabbari [40], the Q-Potts algorithm of Erkizia et al. [41], and lattice schemes developed in adjacent fields for the degradation of heterogeneous three-dimensional media [39]. However, these models have been applied to single-phase materials with static transition probabilities [39,40,41,42] and have not been coupled to phase percolation or composting microbial kinetics. Probabilistic treatments of degradation have also been developed for other polymer systems, such as photochemically induced chain scission in polyethylene glycol [36], highlighting the range of stochastic approaches to polymer degradation beyond the compositions studied here.

Against this backdrop, the present framework combines three-dimensional resolution, Monod-type microbial growth kinetics and composition-dependent enzymatic mechanisms in a single model. It was calibrated against all eight compositions of a complete experimental series, achieving a global RMSE of 3.97% over all fitted points (2.49% excluding two anomalous experimental points), and predicted two compositions withheld from calibration with an RMSE of 3.23%. However, RMSE values should be compared between studies with caution, because they depend on the metric used, the number of data points considered and the composition range. To our knowledge, previous models of polymer-blend biodegradation have not been calibrated across the three degradation regimes (complete, incomplete and minimal) of a full composition series with a single parameter set.

### 4.7. Limitations and Future Directions

The model shows two systematic residuals. First, day-20 disintegration is underestimated by 2–5 points in most blends, while the day-28 value of the 70/30 blend is overestimated by 6.5 points; both depend on the base Monod kinetics that set the start of the erosion front, which cannot be modified without affecting the lag phase and the completion of all compositions simultaneously. Second, the 50/50 blend is overestimated at days 36 and 41 (+5.4 and +5.5 points), because the reachable TPU is consumed at a nearly constant rate rather than decelerating as in the experiment.

The RSA generator idealises the dispersed phase as spherical domains with a log-normal size distribution calibrated to the SEM mean diameters, but it reproduces neither the irregular domain shapes nor the phase inversion reported at 50/50, where PHBV spheres are themselves dispersed within TPU regions [14]. The trapped fraction is derived from the generated TPU network, but the penetration depth *δ* remains a calibrated quantity; deriving it from enzyme diffusion lengths and applying the method to morphologies reconstructed from SEM or micro-computed tomography (µCT) images are natural next steps.

The model does not explicitly consider limitations in oxygen diffusion, changes in pH or the accumulation of soluble products [30,40]. These factors were not required to reproduce the ISO 20200 data, in which the compost is mixed periodically and the pH remains stable, but this does not show that they are negligible. Their relevance is expected to increase under industrial-scale or suboptimal composting conditions, where temperature, moisture and mixing are less tightly controlled [17,18]. Including them would be a natural extension of the present framework.

The present calibration is restricted to a single standardised protocol (ISO 20200). Other methods used to assess the biodegradation of bioplastics under aerobic composting and anaerobic digestion differ in substrate, scale and endpoint criteria [20]. Extending the calibration and validation to these alternative protocols is an area for future research.

Finally, the RMSE of Table 2 quantifies goodness of fit, because the same eight compositions were used to calibrate the parameters and to report the error. The hold-out test provides a first estimate of predictive accuracy within the calibrated composition range (3.23% for the withheld 90/10 and 70/30 blends, compared with 2.70% when they were fitted), but it concerns interior compositions of the same experimental series. Validation against an independent experimental data set (different processing, film thickness or composting protocol) has not been possible with the data currently available and remains necessary before the model can be used for quantitative prediction outside the calibration conditions.

## 5. Conclusions

A three-dimensional, voxel-based Monte Carlo framework for the biodisintegration of PHBV/TPU blends under ISO 20200 composting conditions has been developed, calibrated and tested on withheld compositions. The model incorporates Monod kinetics, coupled biomass and moisture transport, RSA morphology, composition-dependent enzymatic co-degradation and a percolation regime in which the trapped fraction of the TPU phase is derived from the connectivity of the generated TPU network. With 22 adjusted parameters, it achieved a global RMSE of 3.97% over all 40 fitted points (2.49% excluding two anomalous experimental points), with an inter-replicate coefficient of variation of 6.7%, and predicted two compositions withheld from calibration with an RMSE of 3.23%.

The model reproduced three degradation regimes: (i) complete disintegration (≥ 99.5%) by day 36 for blends with ≤ 40 wt% TPU, in which the TPU phase contains no spanning network and is removed by enzymatic co-degradation as the matrix erodes; (ii) incomplete disintegration of the 50/50 blend (~74% simulated, 68% experimental), in which about half of the TPU is trapped within the spanning network, beyond the reach of co-degradation; and (iii) minimal degradation of neat TPU (~10% at day 41), governed by abiotic hydrolysis, with a linear rather than sigmoidal profile. Within the model, the boundary between complete and incomplete disintegration follows from the connectivity of the TPU phase in the generated morphology rather than from a threshold imposed on the kinetics; its location is set by the morphology generator, which follows SEM observations. Sensitivity and identifiability analyses revealed a composition-dependent parameter hierarchy and led to the removal of mechanisms that were not supported by the data. Virtual temperature experiments verified that the thermal correction is consistently implemented (*t*_50_ ∝ 1/*f*(*T*)); experimental tests at other temperatures and against independent data series are required before the framework can be used for the virtual screening of formulations under diverse composting scenarios.

## Figures and Tables

**Table 1:** TPU domain size reported in the literature and random sequential adsorption (RSA; log-normal) parameters used in the model. $\bar{d}$: mean domain diameter measured by scanning electron microscopy (SEM).

Ref.	Processing	PHBV/TPU	$\bar{d}$ (µm)	Morphology	${\bar{r}}_{RSA}$ (vox)	σ_ln_	r_min_–r_max_ (vox)
[14]	Brabender	95/5	0.9	Droplet-in-matrix	2.0	0.4	1.0–4.0
		90/10	1.0	Droplet-in-matrix	2.5	0.4	1.5–5.0
		80/20	2.4	Droplet-in-matrix	3.5	0.5	2.0–6.5
		70/30	3.6	Droplet-in-matrix	4.5	0.5	2.5–8.0
		60/40	3.0	Droplet-in-matrix	5.0	0.6	3.0–9.0
		50/50	(-)	Co-continuous	(-)	(-)	(-)
[15]	Twin-screw ext.	100/10 ^1^	~0.5	Droplet-in-matrix	(-)	(-)	(-)
[13]	Reactive ext.	~77/23 ^2^	0.46	Droplet-in-matrix	(-)	(-)	(-)

^1^ parts per hundred resin (phr) on PHBV. ^2^ 30 phr TPU without hexamethylene diisocyanate (HMDI). The RSA mean radius is prescribed as a monotonic function of TPU content, whereas the measured $\bar{d}$ decreases slightly at 60/40, the composition closest to the co-continuous transition, where a single droplet diameter poorly describes the morphology.

**Table 2:** Simulation (mean of 20 replicates) vs. experimental data [14] as a % disintegration; simulation–experimental error in parentheses. The per-composition and global RMSEs include all points; the two anomalous points (95/5, day 28; 80/20, day 20) are excluded only from the secondary RMSE of 2.49%.

Composition	Day 20	Day 28	Day 34	Day 36	Day 41	RMSE (%)
100/0	4.9 (−0.1)	43.4 (+3.4)	88.6 (−0.4)	100.0 (+0.0)	100.0 (+0.0)	1.51
95/5	5.0 (+0.0)	44.3 (+14.3)	100.0 (+2.0)	100.0 (−0.0)	100.0 (−0.0)	6.45
90/10	4.8 (−2.2)	43.7 (+1.7)	99.5 (+1.5)	99.5 (−0.5)	99.5 (−0.5)	1.46
80/20	4.2 (−13.8)	43.3 (−3.7)	99.7 (−0.3)	99.9 (−0.1)	99.9 (−0.1)	6.37
70/30	3.7 (−4.3)	44.5 (+6.5)	98.9 (+0.9)	100.0 (−0.0)	100.0 (−0.0)	3.53
60/40	3.3 (−4.7)	38.8 (+0.8)	92.6 (−3.4)	99.9 (−0.1)	100.0 (−0.0)	2.64
50/50	2.7 (−3.3)	30.7 (−4.3)	64.3 (+2.3)	70.4 (+5.4)	73.5 (+5.5)	4.32
0/100	0.8 (−0.2)	3.8 (−0.2)	6.6 (−0.4)	7.7 (−0.3)	10.4 (+2.4)	1.10
Global						3.97

**Table 3:** Stochastic variability: RMSE statistics over twenty independent replicates per composition. Domain: 100 × 100 × 20 voxels. SD: standard deviation; CV: coefficient of variation.

Composition	Mean RMSE (%)	±SD (%)	CV (%)	Range (%)
100/0	1.52	0.03	2.0	1.45–1.58
95/5	6.45	0.08	1.2	6.33–6.62
90/10	1.57	0.08	5.3	1.46–1.78
80/20	6.37	0.06	0.9	6.25–6.47
70/30	3.54	0.23	6.4	3.12–3.96
60/40	2.69	0.27	10.1	2.39–3.43
50/50	5.34	1.60	30.0	3.40–8.73
0/100	1.10	0.02	1.5	1.07–1.14
Global	4.16	0.28	6.7	3.86–4.78

**Table 4:** Comparison of nested versions of the model and empirical references (*n* = 40 fitted points). k: number of parameters adjusted to the disintegration data. Hold-out: prediction RMSE for the 90/10 and 70/30 blends withheld from calibration.

Model	*k*	RMSE (%)	RMSE excl. 2 Points (%)	Hold-Out RMSE (%)
M0: no percolation, no neat-PHBV factors	20	8.27	7.85	3.23
M1: M0 + percolation regime	20	4.37	3.12	3.23
M2: final model	22	3.97	2.49	3.23
Earlier version (before removal of non-identifiable mechanisms)	35	3.97	2.53	2.90
Gompertz per composition	24	4.61	—	n/a
Composition-parametric logistic	6	5.26	—	3.68

**Table 5:** Model behaviours that are prescribed by rules, fitted to the data, or emergent from the simulation.

Behaviour	Status	Origin in the Model
Location of the complete/incomplete boundary (40–50 wt% TPU)	Prescribed	Morphology generator: co-continuous at ≥48 wt% TPU, following SEM [14]
Level of the incomplete disintegration of the 50/50 blend	Fitted	Penetration depth *δ* (Equation (18)), calibrated against the 50/50 blend
Absence of trapped TPU in blends with ≤40 wt% TPU	Emergent	No spanning TPU network in the droplet-in-matrix morphologies
Temperature independence of neat-TPU degradation	Prescribed	Hydrolysis (Equation (11)) defined without *f*(*T*)
Start-up and completion of neat PHBV (100/0)	Fitted	Two neat-PHBV factors (0.90 and 0.04)
Sigmoidal acceleration of the blend curves	Fitted	*A* and $a_{thr}$ (Equation (17)), common to all blends
Lag-phase duration (~15–18 days)	Emergent	Inoculum growth (Equations (1–5)) and moisture-gated biomass penetration
Effect of TPU domain size on its removal	Emergent/prescribed	Local exposure on the RSA morphology (Equation (12))/composition-level accessibility (Equation (15))
Spatial patterns: erosion fronts, void networks (Figure 7)	Emergent	Surface-only update, transport and removal
Composition-dependent variability, largest for 50/50 (Table 3)	Emergent	Topology of each generated morphology and kinetic randomness
*t*_50_ ∝ 1/*f*(*T*) scaling of neat PHBV	Largely implied	*f*(*T*) multiplies $\mu_{max}$; the result confirms a consistent implementation rather than an independent prediction

## Data Availability

The complete C source code of the model (the version used to produce all results reported here), the geometry input files, the digitised experimental data (Table A2), and the execution, analysis, Morris-sensitivity and figure-generation scripts are openly available in Zenodo at https://doi.org/10.5281/zenodo.23055744, enabling full reproducibility of the results.

## Appendix A

### Appendix A.1. Complete Parameter Set

Table A1 lists the complete parameter set of the simulation model, grouped by component. The column “Origin” indicates whether each parameter was adjusted to the disintegration data, taken from the literature, measured, fixed a priori or set numerically.

**Table A1:** Parameters of the PHBV/TPU biodisintegration model (final calibration). Origin: C, adjusted to the disintegration data (22 parameters); L, the literature; M, measured (SEM); F, fixed a priori; N, numerical setting.

Parameter	Value	Units	Origin	Description
*µ*_max,PHBV_/*K*_S,PHBV_/*Y*_PHBV_	1.55/0.35/0.25	d^−1^/–/g g^−1^	C	Monod constants of the PHBV phase (Equations (1) and (5))
*µ*_max,TPU_/*K*_S,TPU_/*Y*_TPU_	0.35/0.80/0.005	d^−1^/–/g g^−1^	C	Monod constants of the TPU phase (Equations (1) and (5))
*X*_0_	8 × 10^−10^	–	C	Inoculum on the external faces
*k*_d_/*κ*/*θ*/*K*	0.0003/4.0/0.85/1.0	d^−1^/–/–/–	F	Basal death; carrying-capacity sigmoid (Equations (3) and (4)); K normalises biomass
*b*_0_/*b*_1_/$g_{S}$/$\kappa_{lag}$/*t_lag_*	0.001/0.004/3.0/0.01/15	MCS^−1^/MCS^−1^/–/d^−1^/d	F	Basal PHBV term (Equations (7)–(9))
$\alpha_{MC}$	20.0	–	C	Kinetics-to-probability amplification (Equation (6)
*r*_collapse,PHBV_	0.95	MCS^−1^	C	PHBV collapse rate (Equations (10) and (16))
$S_{c}/$ *X_min_*	0.50/0.01	–	C/F	Collapse substrate threshold/minimum biomass
*A*/$a_{thr}$/*q*	128/0.45/2.0	–	C/C/F	Collapse acceleration (Equation (17))
Neat-PHBV factors	0.90/0.04	–	C	Scale of r_collapse,PHBV_ and of A for 100/0 only
*p*_hyd_/*κ*_lag,TPU_/*t*_lag,TPU_	0.012/2.0/15	MCS^−1^/d^−1^/d	C	Abiotic hydrolysis of TPU (Equation (11))
*m*	1.2	–	C	Exponent of local accessibility (Equation (12))
$r_{codeg}$	1.8	MCS^−1^	C	Global co-degradation constant (Equation (14))
*p*/*λ*_E_	2.0/0.95	–/– (per MCS)	F	Front-advance exponent (Equation (14))/enzyme retention
Accessibility reference	0.35	–	C	TPU fraction at which f_accessibility_ saturates (Equation (15))
*δ*	2	vox	C	Penetration depth into the spanning TPU network (Equation (18))
Cross-affinity	0.10	–	C	Biomass transfer across a PHBV/TPU interface
$\alpha_{diff}/p_{Y}(moisture,biomass)$/moisture threshold	0.12/0.98, 0.90/0.2	–	F	Transport (Equation (19)); biomass moves only into voxels with moisture above the threshold
Inheritance fraction/weights	0.8/1.0, 0.05	–	F	Biomass redistribution on removal (PHBV, TPU)
Integrity decrements	10/128	8-bit units	N	PHBV/TPU
Initial moisture/moisture after removal	0.9/1.0	-	N	Moisture of the inoculated faces at *t* = 0/of a vacated site
*T*_min_/*T*_opt_/*T*_max_	5/58/70	°C	L [44]	Cardinal temperatures (CTMI)
Δ*t*	0.16	d	N	Time per MCS
RSA radius/*σ*_ln_/separation	2.0 → 5.0/0.4 → 0.6/1	vox/-/vox	M [13,14,15]	Morphology by composition (Table 1)
Co-continuous generator	≤0.52/8/0.05–0.35	–/–/rad vox^−1^	M [14]/N/N	PHBV fraction below which it is used/number of waves/frequency range

### Appendix A.3. Experimental Reference Data

**Table A2:** Disintegration (%) of PHBV/TPU blends under ISO 20200 at 58 °C, digitised from Figure 6 of Martínez-Abad et al. [14] (estimated digitisation uncertainty ±1 percentage point).

Day	100/0	95/5	90/10	80/20	70/30	60/40	50/50	0/100
0	0	0	0	0	0	0	0	0
6	0	0	0	0	0	0	0	0
13	0	0	0	0	0	0	0	0
20	5	5	7	18	8	8	6	1
28	40	30	42	47	38	38	35	4
34	89	98	98	100	98	96	62	7
36	100	100	100	100	100	100	65	8
41	100	100	100	100	100	100	68	8
